# Dual-RNA-sequencing to elucidate the interactions between sorghum and *Colletotrichum sublineola*

**DOI:** 10.3389/ffunb.2024.1437344

**Published:** 2024-08-16

**Authors:** Saddie Vela, Emily S. A. Wolf, Jeffrey A. Rollins, Hugo E. Cuevas, Wilfred Vermerris

**Affiliations:** ^1^ Plant Molecular & Cellular Biology Graduate Program, University of Florida, Gainesville, FL, United States; ^2^ Department of Plant Pathology, University of Florida, Gainesville, FL, United States; ^3^ United States Department of Agriculture, Agricultural Research Service, Tropical Agriculture Research Station, Mayagüez, PR, United States; ^4^ Department of Microbiology & Cell Science, University of Florida, Gainesville, FL, United States; ^5^ University of Florida Genetics Institute, Gainesville, FL, United States

**Keywords:** anthracnose, defense, effector, hemibiotroph, immunity, *Sorghum bicolor*, transcriptomics

## Abstract

In warm and humid regions, the productivity of sorghum is significantly limited by the fungal hemibiotrophic pathogen *Colletotrichum sublineola*, the causal agent of anthracnose, a problematic disease of sorghum (*Sorghum bicolor* (L.) Moench) that can result in grain and biomass yield losses of up to 50%. Despite available genomic resources of both the host and fungal pathogen, the molecular basis of sorghum−*C. sublineola* interactions are poorly understood. By employing a dual-RNA sequencing approach, the molecular crosstalk between sorghum and *C. sublineola* can be elucidated. In this study, we examined the transcriptomes of four resistant sorghum accessions from the sorghum association panel (SAP) at varying time points post-infection with *C. sublineola*. Approximately 0.3% and 93% of the reads mapped to the genomes of *C. sublineola* and *Sorghum bicolor*, respectively. Expression profiling of *in vitro* versus *in planta C. sublineola* at 1-, 3-, and 5-days post-infection (dpi) indicated that genes encoding secreted candidate effectors, carbohydrate-active enzymes (CAZymes), and membrane transporters increased in expression during the transition from the biotrophic to the necrotrophic phase (3 dpi). The hallmark of the pathogen-associated molecular pattern (PAMP)-triggered immunity in sorghum includes the production of reactive oxygen species (ROS) and phytoalexins. The majority of effector candidates secreted by *C. sublineola* were predicted to be localized in the host apoplast, where they could interfere with the PAMP-triggered immunity response, specifically in the host ROS signaling pathway. The genes encoding critical molecular factors influencing pathogenicity identified in this study are a useful resource for subsequent genetic experiments aimed at validating their contributions to pathogen virulence. This comprehensive study not only provides a better understanding of the biology of *C. sublineola* but also supports the long-term goal of developing resistant sorghum cultivars.

## 1 Introduction

**Figure 1:**
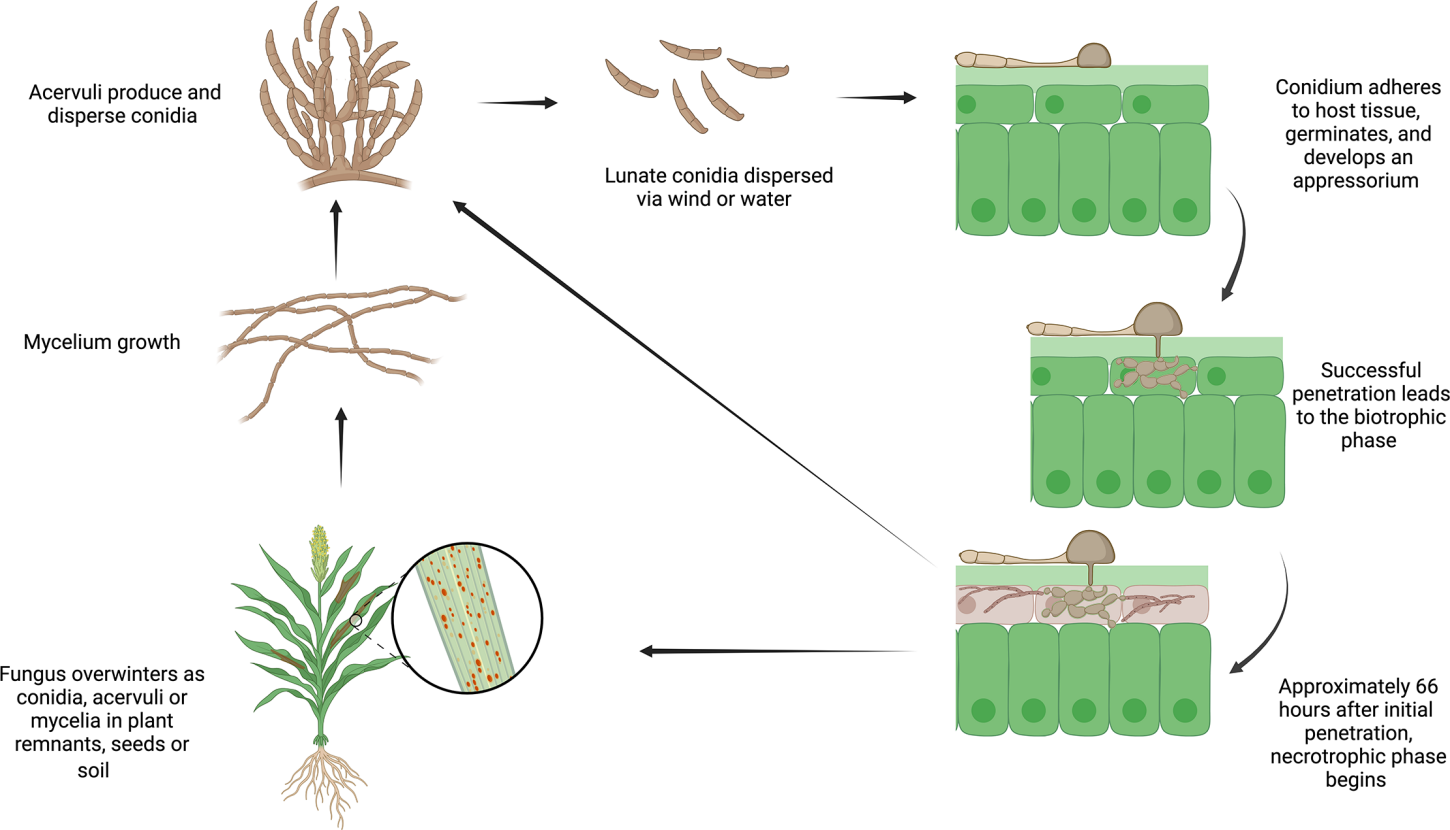
The life cycle of *Colletotrichum sublineola*, adapted from Crouch and Beirn (2009). Figure created with BioRender.com.

*Colletotrichum sublineola* (Henn.) in Kabat & Bubak, the causative agent of anthracnose in sorghum *(Sorghum bicolor* (L.) Moench), is a hemibiotrophic pathogen. It is characterized by initially infecting living host tissue (biotrophic phase) and subsequently transitioning to killing and feeding on dead host tissue (necrotrophic phase) (**Figure 1**). *C. sublineola* infection of sorghum leaves manifests itself by the appearance of lesions with dark red or tan margins (Pastor-Corrales and Frederiksen, 1978). The necrotic parts of the surface contain acervuli that display dark setae (protruding specialized hyphae with thick melanized walls) (Pastor-Corrales and Frederiksen, 1978). The progression of anthracnose into the stem gives rise to red rot, which is characterized by the appearance of red marbling in the stalk and subsequently weakens the stem’s integrity (Lebeau et al., 1951; Harris et al., 1964; Tesso et al., 2012). In the later stages of development, infection can be observed in the rachis, panicle branches, and seeds, leading to a direct reduction in grain yield (Harris et al., 1964). Efficient development of resistant cultivars and effective disease management strategies against anthracnose benefit from a comprehensive understanding of the interactions between sorghum and *C. sublineola*, which is presently incomplete (Stutts and Vermerris, 2020).

The evolutionary “arms race” between plants and pathogens is characterized by a continual battle for survival and adaptation (Jones and Dangl, 2006). This interaction is characterized by the initiation of a pattern-triggered immune response (PTI) upon the recognition of pathogen/damage-associated molecular patterns (PAMPs/DAMPs) (Jones and Dangl, 2006; Win et al., 2012). Conversely, pathogens can overcome this defense layer by releasing effectors that facilitate the colonization of the host. Effectors are proteins that disrupt the structure or processes of the host cell, suppressing defense responses and increasing access to nutrients (Win et al., 2012). Detection of pathogen effectors by receptors encoded by the host’s resistance (*R*) genes activates the second defense layer, known as effector-triggered immunity (ETI), which may lead to a hypersensitive reaction (HR) (Win et al., 2012). To date, there have been several studies reporting sorghum’s resistance responses in different genotypes that demonstrated the existence of multiple resistance mechanisms (reviewed by Stutts and Vermerris, 2020 and Abreha et al., 2021). However, the identification and characterization of key molecular determinants of *C. sublineola* pathogenicity have yet to be elucidated.

Past studies on the diversity of *C. sublineola* pathotypes uncovered differences in diversity that are dependent on year, location, and/or presence of dominant haplotypes (Rosewich et al., 1998; Valèrio et al., 2005; Thakur et al., 2007; Moore et al., 2008; Prom et al., 2024). The high variability and genetic diversity of *C. sublineola* make it challenging to breed sorghum cultivars that are reliably resistant across years and locations. To enhance sorghum resistance against the pathogen, continuous improvements in genome annotation, re-sequencing of various host genotypes and pathogen strains, and functional genomics approaches are crucial. The availability of genome sequences for various pathotypes of *C. sublineola* enables further genome sequencing and transcriptomics studies to identify expressed genes during different infection stages (Baroncelli et al., 2014; Buiate et al., 2017; Baldrich et al., 2021).

In a notable comparative genomic study, gene models from two strains of the closely related species *C. graminicola* and *C. sublineola*, pathogens of maize and sorghum, respectively, were analyzed to identify genes that are not conserved, indicating potential candidates for involvement in host specificity (Buiate et al., 2017). The genomes of *C. graminicola* M1.001 and *C. sublineola* CgSl1 harbored approximately 1,000 genes encoding predicted proteins that were unique to each species, termed non-conserved proteins (NCPs). A majority (>65%) of the NCPs in both strains did not match any conserved protein family (Pfam) categories. Among the minority of NCPs with Pfam classifications, the largest groups consisted of transporters, cytochrome P450s, secondary metabolite-associated proteins, carbohydrate-active enzymes (CAZymes), and transcription factors. This highlights the importance of adapting to diverse aspects of each host environment and in the secretion or evasion of toxic secondary metabolites (Buiate et al., 2017).

While significant progress has been made in recent years regarding disease resistance loci and defense mechanisms, our understanding of the interactions between sorghum and *C. sublineola* remains incomplete. Advances in genomics, transcriptomics, proteomics, and metabolomics studies can help elucidate the complex molecular crosstalk during sorghum–*C. sublineola* interactions (Stutts and Vermerris, 2020). However, it is only recently that transcriptomics (Gan et al., 2013; Buiate et al., 2017; Wang et al., 2020; Wolf et al., 2024) and metabolomics (Tugizimana et al., 2019) approaches have started to be applied to study the interactions between sorghum and *C. sublineola*.

Dual-RNA-sequencing offers a comprehensive insight into host–pathogen interactions (Westermann et al., 2012). Tierney et al. (2012) reported the first successful application of dual RNA-seq to a eukaryotic interaction model between *Candida albicans* and dendritic cells in its mouse host. This approach has also been utilized in sorghum–*Bipolaris sorghicola* interaction via *de novo* assembly of the pathogen transcriptome to identify key genes in the plant–pathogen interaction (Yazawa et al., 2013). Here, we present a dual RNA-sequencing approach to examine the interaction between different sorghum genotypes and *C. sublineola* at the molecular level at different stages of infection. Furthermore, the infection process was observed *in planta* with epi-fluorescence microscopy.

## 2 Materials and methods

### 2.1 *Colletotrichum sublineola* growth conditions and infection in sorghum for RNA-sequencing

*C. sublineola* isolates were collected from sorghum leaf tissue at the UF North Florida Research and Education Center-Suwannee Valley near Live Oak, FL (30.313277 N, 82.902158 W) according to Felderhoff et al. (2016). Isolates were cultured on potato dextrose agar (PDA) at room temperature for 10 to 14 days. To create a conidial suspension, 1–5 mL of sterile ddH_2_O was added to the Petri dish, and the agar surface was gently agitated using a sterile L-shaped spreader. The conidial concentration in the suspension was determined using a hemocytometer and adjusted to a final concentration of 10^6^ conidia/mL. A mock solution was prepared using sterile water. Both the conidial and mock solutions were supplemented with three drops of Tween-20 (Sigma-Aldrich, St Louis, MO) for a final volume of 5 mL and stirred at low speed for 10 minutes. Subsequently, these solutions were transferred into spray bottles according to the respective treatments.

The sorghum accessions SC17, SC110, SC1033, and SC1330 were selected for analysis because they have different mechanisms to combat anthracnose. Accessions SC17, SC110, and SC1330 have resistance alleles for one of the three anthracnose-resistance loci on chromosome 5 (**Supplementary Table 1**), whereas SC1033 is susceptible at all three loci but may have resistance alleles at other loci (Cuevas et al., 2018). Since anthracnose resistance is a multigenic trait, these alleles offer only partial resistance and do not entirely prevent disease (**Supplementary Figure 1**). Accessions were cultivated in a greenhouse in a split-split plot design for accession and time points (1, 3, 5 dpi) with four biological replicates (*n* = 4 per time point) as described by Wolf et al. (2024). Mock-inoculated and inoculated treatments were grown in separate plastic tents to prevent contamination of the control treatment. At the fifth-leaf stage, plants were sprayed with 1 mL of the conidial suspension or the mock-inoculation solution.

### 2.2 Isolation of fungal and plant RNA and library preparation for RNA-sequencing

RNA was extracted from *C. sublineola* cultured for two weeks on PDA plates with a nylon membrane (2 µm) overlay (Sigma-Aldrich, St. Louis, MO) and fungal samples were subjected to two chloroform extractions prior to RNA extraction as described by Schumann et al. (2013). Mock-inoculated control and inoculated leaf tissue samples were collected from all four sorghum genotypes at 1, 3, and 5 dpi. Tissue was immediately frozen in liquid nitrogen and homogenized with a BeadBlaster24 (Benchmark Scientific, Sayreville, NJ) and subjected to RNA extraction as described by Wolf et al. (2024). Approximately 1–3 μg of total RNA per sample was sent to Novogene, Inc. (Sacramento, CA) for library preparation and paired-end sequencing with the Illumina NovaSeq 6000 PE150 sequencing platform. The transcripts obtained from *C. sublineola* cultured on PDA plates were referred to as *in vitro* transcripts and served as a reference for the transcripts obtained from *C. sublineola* growing in infected sorghum tissue, which were referred to as *in planta* transcripts.

### 2.3 Bioinformatics analysis

Raw reads were processed with *FASTQC* (v0.11.7) to remove low-quality reads (Andrews, 2010). *Trimmomatic* (v.0.35) was used to eliminate adapter sequences and to improve read quality (Bolger et al., 2014). Reads with a Phred score > 20 and read length > 50 bp were selected for downstream analysis (Corchete et al., 2020). The subsequent trimmed and filtered pair-end reads were aligned to the BTx623 sorghum reference genome v. 3.1 (Paterson et al., 2009; McCormick et al., 2018) and the *C. sublineola* reference genome (Baroncelli et al., 2014) using *HiSat2* (v2.2.1) (Kim et al., 2015). The number of reads aligned was counted using *HTSeq* (v.0.11.2) in combination with *EdgeR*’s (v3.28.0) trimmed mean of M values (TMM) to normalize the data for effective library size with TMM normalization, RNA composition, sequencing depth, and corrected for multiple testing (Robinson et al., 2010; Anders et al., 2015). A generalized linear model (GLM) quasi-likelihood F-test with a false discovery rate (FDR) of 0.05 was used to identify differentially expressed genes (DEGs) (Benjamini and Hochberg, 1995).

### 2.4 KEGG enrichment analysis for differentially expressed sorghum genes

KEGG Orthology Based Annotation System (KOBAS) 2.0 was used for enrichment analysis of the differentially expressed genes (Bu et al., 2021). KOBAS offers pathway enrichment analysis by assigning orthology to input genes and maps them to Kyoto Encyclopedia of Genes and Genomes (KEGG) pathways (Kanehisa et al., 2004). A Fisher’s exact test was used to determine the significantly enriched pathways while controlling the FDR (Benjamini and Hochberg, 1995). Significantly enriched pathways were determined based on adjusted p-values or q-values below a predefined threshold (FDR < 0.05).

### 2.5 Gene ontology annotation of fungal up-regulated genes

Significantly up-regulated genes at the different sampling time points were assigned Gene Ontology (GO) (Gene Ontology Consortium, 2021) terms using BLAST2GO (Götz et al., 2008) in OmicsBox software (OmicsBox, 2019). BLASTP (Altschul et al., 1990) was utilized to identify amino acid sequences with similarities to the query sequences in the NCBI protein sequence database with an e-value cutoff of 1 × 10^−3^ (Buchfink et al., 2021), then GO mapping was used to retrieve GO terms associated with the BLAST hits (Ashburner et al., 2000; Götz et al., 2008; OmicsBox, 2019). GO annotations were assigned to the query sequences based on the GO mapping results using default filters (Ashburner et al., 2000; Götz et al., 2008; OmicsBox, 2019). These results were categorized using GO-Slim to summarize GO annotations (Götz et al., 2008; OmicsBox, 2019). For each time point comparison, BLAST2GO was used to calculate the abundance of GO classifications for query sequences of the genes that were statistically significantly up-regulated.

### 2.6 Fungal effector prediction

Effector proteins were predicted using a streamlined bioinformatics analysis according to the procedure of Lu et al. (2022). First, predicted proteins were analyzed for the presence of signal peptides with SignalP 5.0 (Almagro Armenteros et al., 2019) and extracellular localization with WoLF-PSORT (Horton et al., 2007). Next, DeepTMHMM (Hallgren et al., 2022) and PredGPI (Pierleoni et al., 2008) were used to exclude proteins with transmembrane helices and glycosylphosphatidylinositol (GPI) anchors, respectively. EffectorP 3.0 was used to identify and categorize predicted effector proteins secreted into the apoplast, cytoplasm, or both (Sperschneider and Dodds, 2022).

### 2.7 Characterization of putative effectors and structural network analysis

The amino acid sequences of annotated and unannotated (no signal peptide, no transmembrane domain, no assigned function) putative effector proteins were analyzed with SignalP v5.0 (Almagro Arementeros et al., 2019) to identify predicted cleavage sites and N-terminal signal peptides. All subsequent analyses were performed on effector sequences lacking their signal peptide. Nuclear localization sequences (NLS) were predicted for all effectors with NLStradamus (Nguyen Ba et al., 2009). Structural modeling of effectors was performed with AlphaFold2 (Jumper et al., 2021; Mirdita et al., 2022). A tolerance of 0.5 was employed for alphafold2_ptm monomer prediction, and models were ranked based on predicted LDDT (pLDDT) scores to determine the best model. The top-ranked AlphaFold model was utilized for protein structural network construction and FoldSeek analyses (van Kempen et al., 2024). Protein structural networks were constructed as described by Teulet et al. (2023), using a template modeling (TM)-score threshold of 0.6. Protein structural networks were visualized with the R package IGRAPH employing the Louvain community detection method (Csárdi et al., 2006; Blondel et al., 2008).

### 2.8 Identification of fungal proteins predicted to be carbohydrate-active enzymes

Identification of carbohydrate-active enzymes (CAZymes) was performed using dbCAN3 (https://bcb.unl.edu/dbCAN2/) (Zheng et al., 2023). The dbCAN3 web server provides Hidden Markov Models of Emission for Recognition (HMMER) search of the dbCAN HMM database (*e*-value <1 × 10^–15^ and coverage >0.35) (Finn et al., 2011), and a DIAMOND (Buchfink et al., 2015) search of the CAZy pre-annotated CAZyme sequence database (*e*-value <10^–102^). CAZyme families have been defined and classified by the CAZy database (Lombard et al., 2014), forming six major classes: glycosyltransferases (GTs), glycoside hydrolases (GHs), polysaccharide lyases (PLs), carbohydrate esterases (CEs), carbohydrate-binding modules (CBM) and enzymes for the auxiliary activities (AAs).

### 2.9 Classification of membrane transporters

Predicted membrane transporters were classified based on the GO annotations (Götz et al., 2008) related to transmembrane transport (GO:0055085) and transporter activity (GO:0005215).

### 2.10 Pathogen-Host Interaction database

To identify similarities to proteins indicative of known pathogenicity and virulence factors present in the Pathogen-Host Interaction (PHI) database (www.phi-base.org) (Winnenburg et al., 2006), predicted *C. sublineola* protein sequences were used as queries in PHIB-BLAST (http://phi-blast.phi-base.org/) (Urban et al., 2019) version 4.16 with an e-value cutoff of 1 × 10^−5^.

### 2.11 Identification of biosynthetic gene clusters

Biosynthetic gene clusters (BGCs) in *C. sublineola* were identified using antiSMASH version 7.1.0 for fungi (Blin et al., 2023). Genome sequences of interest were uploaded by NCBI accession number and BGCs were detected using relaxed detection strictness. ClusterBLAST (Blin et al., 2023) was utilized to determine similar BGCs in other fungal pathogens.

### 2.12 Aniline blue staining of infected sorghum leaf tissue

SC1033 plants were grown in a greenhouse and leaves were collected at the fifth-leaf stage. Leaves were cut into sections (7.5 × 2.5 cm^2^), placed in a Petri dish, and drop-inoculated using a syringe with 1 mL of a conidial suspension prepared as described previously. Leaf tissue (*n* = 3) was collected for fixation and staining at 1-, 3-, and 5 days post-inoculation based on the protocol by Bhadauria et al. (2010) and Hood and Shew (1996). At each time point, leaf tissue was placed in a fixative solution consisting of 60% (v/v) methanol, 30% (v/v) chloroform, and 10% (v/v) glacial acetic acid until required for staining. Leaf samples were rehydrated by immersion in an ethanol series with decreasing concentrations (100, 80, 70, and 50 (v/v) % in water). Leaf tissue was then placed in 1M KOH solution in individual 1.5 mL microcentrifuge tubes and incubated in a thermoblock (Benchmark Scientific, Sayreville, NJ) at 100°C for 15 minutes. The leaf tissue was washed three times for 15 minutes with deionized water. Samples were stained with 0.05% (w/v) aniline blue (Sigma-Aldrich, St Louis, MO) in 150 mM K_2_HPO_4_ at pH 9.5 overnight. Leaves were then de-stained in 150 mM K_2_HPO_4_ at pH 9.5, mounted on a microscope slide, and covered with a cover slip. The infection process was observed using a Leica DMR compound epi-fluorescence microscope (Leica Microsystems, Wetzlar, Germany) with a narrow UV long pass (LP) filter set (355–375 nm excitation, and 400LP nm emission). Images were captured with a Leica DFC450 C CCD camera using Leica Application Suite X software v.4.8.

## 3 Results

### 3.1 RNA-Seq mapping statistics of reads mapped to the sorghum and *C. sublineola* genomes

To elucidate the sorghum responses to *C. sublineola* in inoculated leaf tissue and to profile the gene expression dynamics of *C. sublineola* throughout the infection process, we performed a dual RNA-seq approach on tissue samples collected at three time points. Approximately 42 million reads obtained from individual inoculated and mock-inoculated samples were mapped to the *Sorghum bicolor* BTx623 reference genome v3.1.1 (Paterson et al., 2009; McCormick et al., 2018) (**Supplementary Data 1**). Additionally, an average of 126,000 reads were mapped to the *C. sublineola* genome (Baroncelli et al., 2014) from *in planta* transcripts (**Supplementary Data 1**). This meant that, on average, 93% of the reads from inoculated and mock-inoculated samples mapped to the sorghum genome, and 0.3% of reads mapped to the *C. sublineola* genome (**Supplementary Data 1**), the difference reflective of the fact that the fungal cells were far outnumbered by plant cells in the leaf tissue used for RNA extraction. In parallel to the *in planta* analysis, transcriptomic data were gathered from the *C. sublineola* isolate cultivated *in vitro*. This dataset served as a reference to delineate differences in expression relative to growth *in planta* (**Supplementary Data 1**).

**Figure 2:**
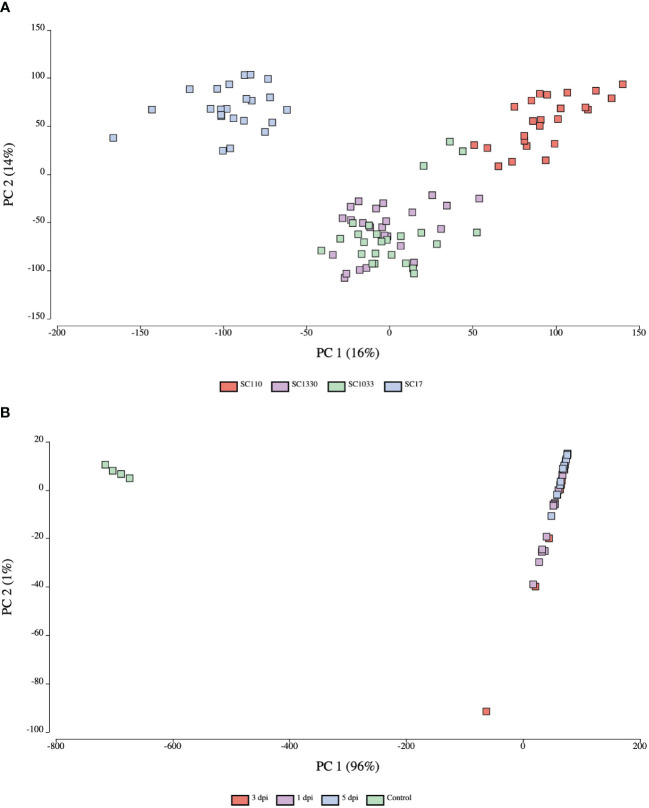
Principal component score plots of expression profiles from host and pathogen samples. **(A)** Sorghum samples clustered by genotype: SC17 (blue), SC110 (red), SC1033 (green), and SC1330 (purple). **(B)** *C. sublineola* samples clustered by *in planta* (inoculated) and *in vitro* (control) (green) samples. Inoculated samples were collected at 1 dpi (purple), 3 dpi (red), and 5 dpi (blue). Figures created in OmicsBox.

To analyze the relatedness of the biological replicates and gene expression patterns between samples from both the host and pathogen, a principal component analysis (PCA) was conducted using OmicsBox. The PC score plot of sorghum samples revealed distinct gene expression patterns based on genotype (**Figure 2A**). The first two principal components (PC1 and PC2) explained 16% and 14% of the total variance in the dataset, respectively. The PCA of *C. sublineola* transcripts indicated distinct gene expression patterns in *in vitro* (control) and *in planta* (inoculated) samples (**Figure 2B**). Almost all of the variance (96%) in the dataset was captured in PC1. *In planta* samples from different time points overlapped in the PC score plot (**Figure 2B**; **Supplementary Figure 2**). Notably, the overlap of 3 dpi samples with 1 dpi and 5 dpi samples indicated overall similar expression profiles. The larger library size of one replicate at 3 dpi likely caused it to be an outlier. The expression profile of the *in planta* samples by time point explained 43% of the variance (PC1) (**Supplementary Figure 2**).

### 3.2 Differentially expressed genes in sorghum in response to infection

**Figure 3:**
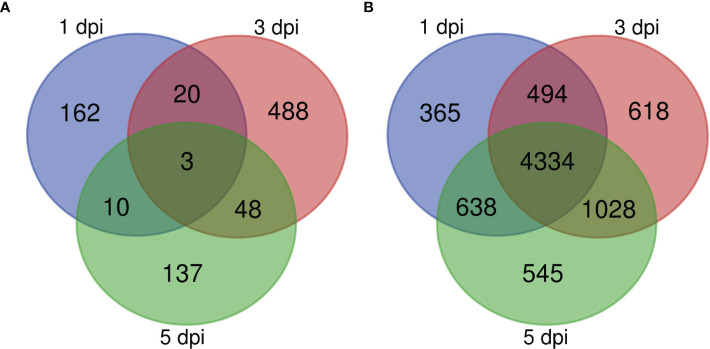
Venn diagram showing the numbers of DEGs identified in **(A)** sorghum and **(B)** *C. sublineola* transcriptomes at 1, 3, and 5 dpi. Figure made with the tool available at: https://bioinformatics.psb.ugent.be/webtools/Venn/.

**Figure 4:**
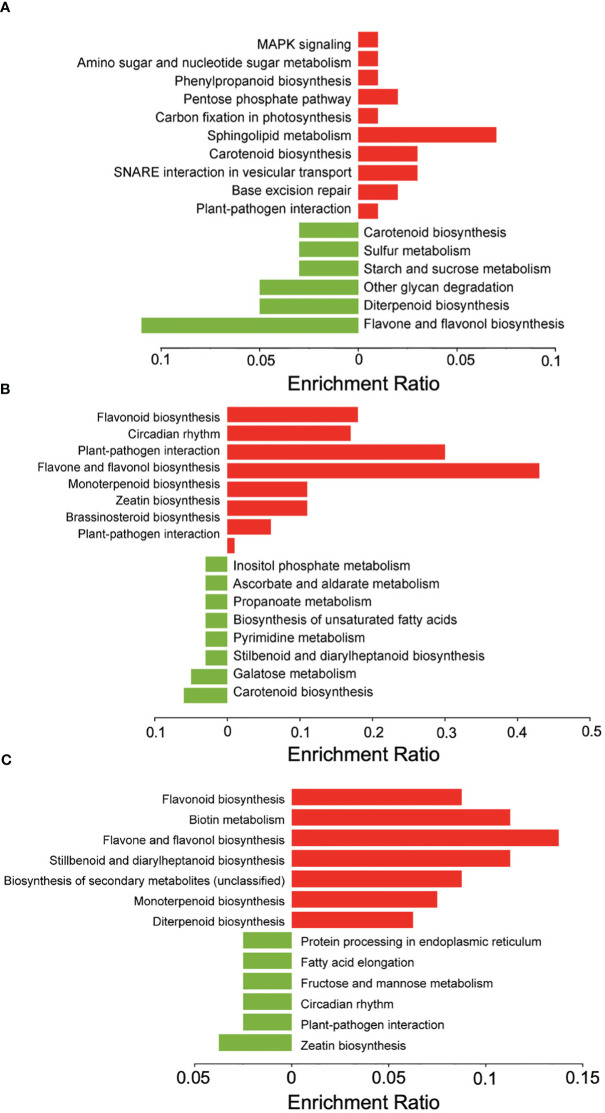
KEGG pathway enrichment analysis of DEGs in sorghum. Down-regulated genes (green) and upregulated genes (red) at **(A)** 1 dpi, **(B)** 3 dpi, and **(C)** 5 dpi in inoculated versus mock-inoculated samples. Figure created with SRPlot (Tang et al., 2023).

An analysis of differentially expressed genes in inoculated samples compared to mock-inoculated controls across all genotypes at 1, 3, and 5 dpi revealed that at 1 dpi a total of 93 genes were up-regulated and 102 were down-regulated, and that 162 of the total differentially expressed genes (DEGs) were unique to 1 dpi (**Figure 3A**). KEGG pathway enrichment of the up-regulated genes at 1 dpi revealed pathways enriched for sphingolipid metabolism, carotenoid biosynthesis, and SNARE interactions in vesicle transport (**Figure 4A**). At 3 dpi there were 343 up-regulated and 216 down-regulated genes, and 488 of the total DEGs were uniquely expressed at 3 dpi (**Figure 3A**). Enriched biological pathways represented by up-regulated genes at 3 dpi included flavonoid biosynthesis, specifically the biosynthesis of flavones and flavonols (**Figure 4B**). At 5 dpi a total of 139 genes were up-regulated, 59 were down-regulated, and 137 genes were uniquely expressed at 5 dpi (**Figure 3A**). KEGG pathway enrichment of the up-regulated DEGs at 5 dpi identified genes involved in biotin metabolism and the biosynthesis of flavonoids (specifically flavones and flavonols), stilbenoids, diarylheptanoids and various other secondary metabolites (**Figure 4C**).

### 3.3 Differentially expressed *C. sublineola* genes during infection

**Figure 5:**
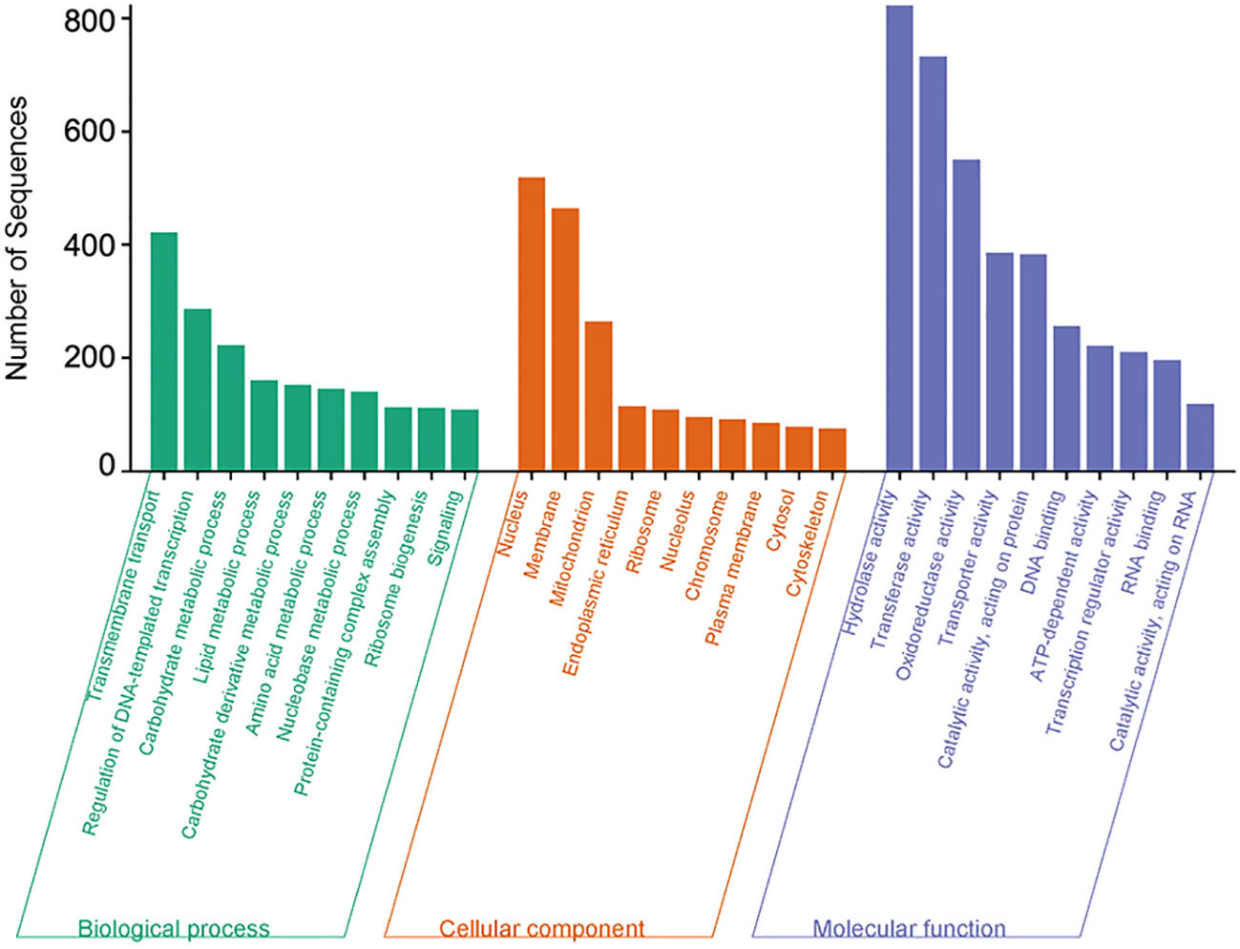
Overrepresented Gene Ontology (GO) terms of *C. sublineola* DEGs in inoculated versus control samples. Figure created with SRPlot (Tang et al., 2023).

The comparison between *in planta* (inoculated) and *in vitro* (control) *C. sublineola* samples revealed that the vast majority of DEGs were expressed at lower levels *in planta*: 7,469 genes versus only 459 genes with higher expression during infection. It is likely that the disproportionally large differences in DEGs are due to the substantial difference between the number of *C. sublineola* reads *in vitro* versus *in planta* samples. Gene Ontology (GO) annotation indicated that most DEGs had molecular functions related to hydrolase, transferase activity, oxidoreductase, protein catalytic and transporter activity, as well as DNA binding (**Figure 5**). Biological processes represented by these DEGs were transmembrane transport, regulation of transcription, and carbohydrate, lipid, and amino acid metabolism (**Figure 5**). These results indicated a lack of nutrient availability *in planta* caused the low expression of genes involved in energy-related metabolism, nutrient acquisition, and growth in an attempt to conserve resources and adapt to the host environment.

Expressed genes in *C. sublineola* were compared between time points (1, 3, 5 dpi) to identify genes important for infection during the transition from the biotrophic phase (1 dpi) to the necrotrophic phase (5 dpi). At 1 dpi 238 genes were up-regulated, and 5,593 genes were down-regulated. Among the collective set of fungal DEGs, 365 were unique to 1 dpi (**Figure 3B**). At 3 dpi a total of 411 up-regulated genes and 6,063 down-regulated genes were identified and 618 of the total DEGs were unique to 3 dpi (**Figure 3B**). At 5 dpi 158 up-regulated genes and 6,387 down-regulated genes were identified and 545 genes were expressed uniquely at this timepoint (**Figure 3B**).

**Figure 6:**
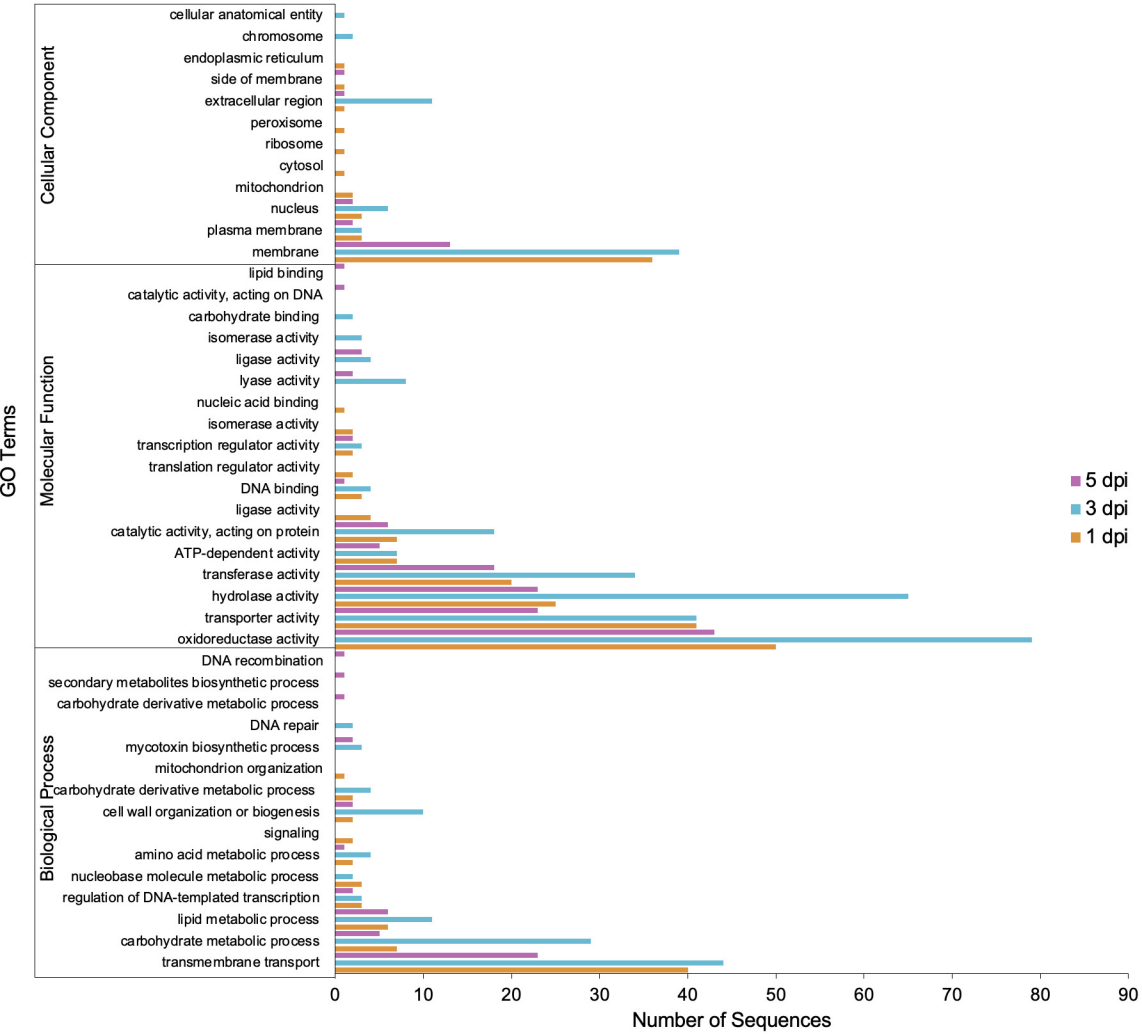
Gene Ontology (GO) terms associated with *C. sublineola* genes up-regulated in the *in planta* versus *in vitro samples* by timepoint.

**Figure 7:**
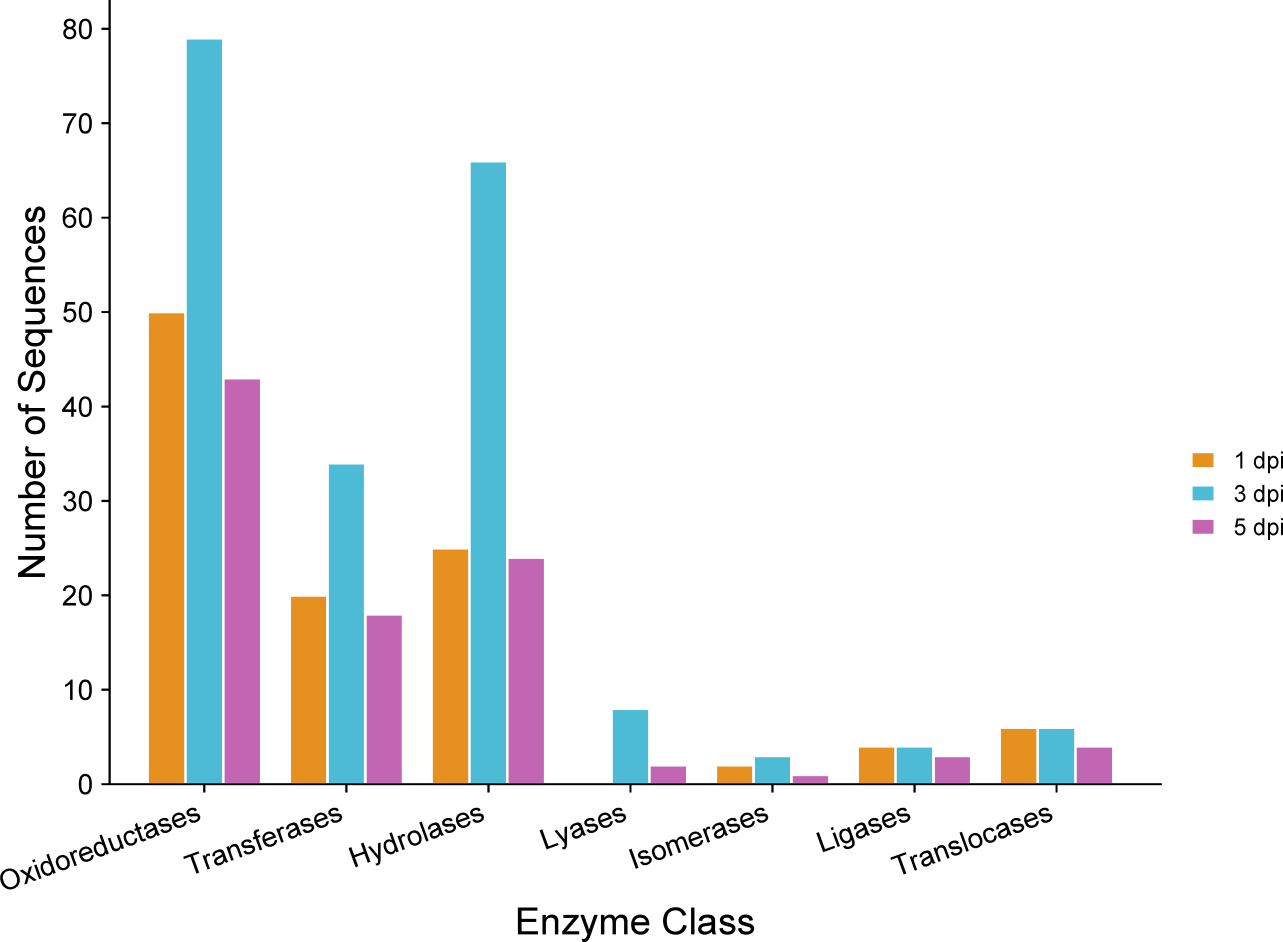
Gene Ontology (GO) of enzymes encoded by *C. sublineola* genes up-regulated *in planta* versus *in vitro samples* at different time points. Figure created with SRPlot (Tang et al., 2023).

GO annotation for up-regulated genes at different time points during infection revealed an overrepresentation of biological processes related to transmembrane transport, carbohydrate metabolism, and lipid metabolism (**Figure 6**). Subsequently, the proteins encoded by the up-regulated genes were predicted to be primarily located in the membrane (**Figure 6**). Molecular processes of up-regulated genes were associated with oxidoreductase, hydrolase, transferase, and transport activities (**Figure 6**). Thus, among the enzymes encoded by up-regulated genes, oxidoreductases and hydrolases were the most abundant classes (**Figure 7**). Analysis of the up-regulated genes at 3 dpi implicated that distinct processes were occurring at this time point compared to the other two time points (1 and 5 dpi), including cell wall biogenesis, which includes cell wall organization and secretion of proteins into the extracellular space (**Figure 6**).

In genotype SC17, 90 C*. sublineola* genes were up-regulated and 5,121 were down-regulated relative to *C. sublineola* cultured *in vitro*. In SC110, 216 genes were up-regulated and 5,772 down-regulated, whereas in SC1330 there were 484 up-regulated genes and 3,995 down-regulated genes. In accession SC1033, which lacked resistance alleles at the three candidate resistance loci on chromosome 5, 99 C*. sublineola* genes were up-regulated and 6,742 down-regulated.

An analysis of the up-regulated *C. sublineola* genes shared between sorghum accessions revealed that SC1330 displayed the highest number of unique up-regulated genes (**Supplementary Figure 3**). The different expression patterns imply that *C. sublineola* deployed different strategies to infect sorghum genotypes with different resistance mechanisms.

### 3.4 Carbohydrate-active enzymes deployed by *C. sublineola* to facilitate infection

Carbohydrate-active enzymes (CAZymes) are involved in various crucial biological processes, including cell wall biogenesis, signaling, and energy production (Park et al., 2010). The CAZymes of classes carbohydrate esterases (CE), glycoside hydrolases (GH), and polysaccharide lyases (PL) are often known as cell wall degrading enzymes (CWDEs) because they play pivotal roles in the decomposition of plant cell walls (PCW) (Ospina-Giraldo et al., 2010). Profiling the expression of genes encoding different classes of CAZymes during infection can reveal strategies used to degrade the host cell wall.

**Figure 8:**
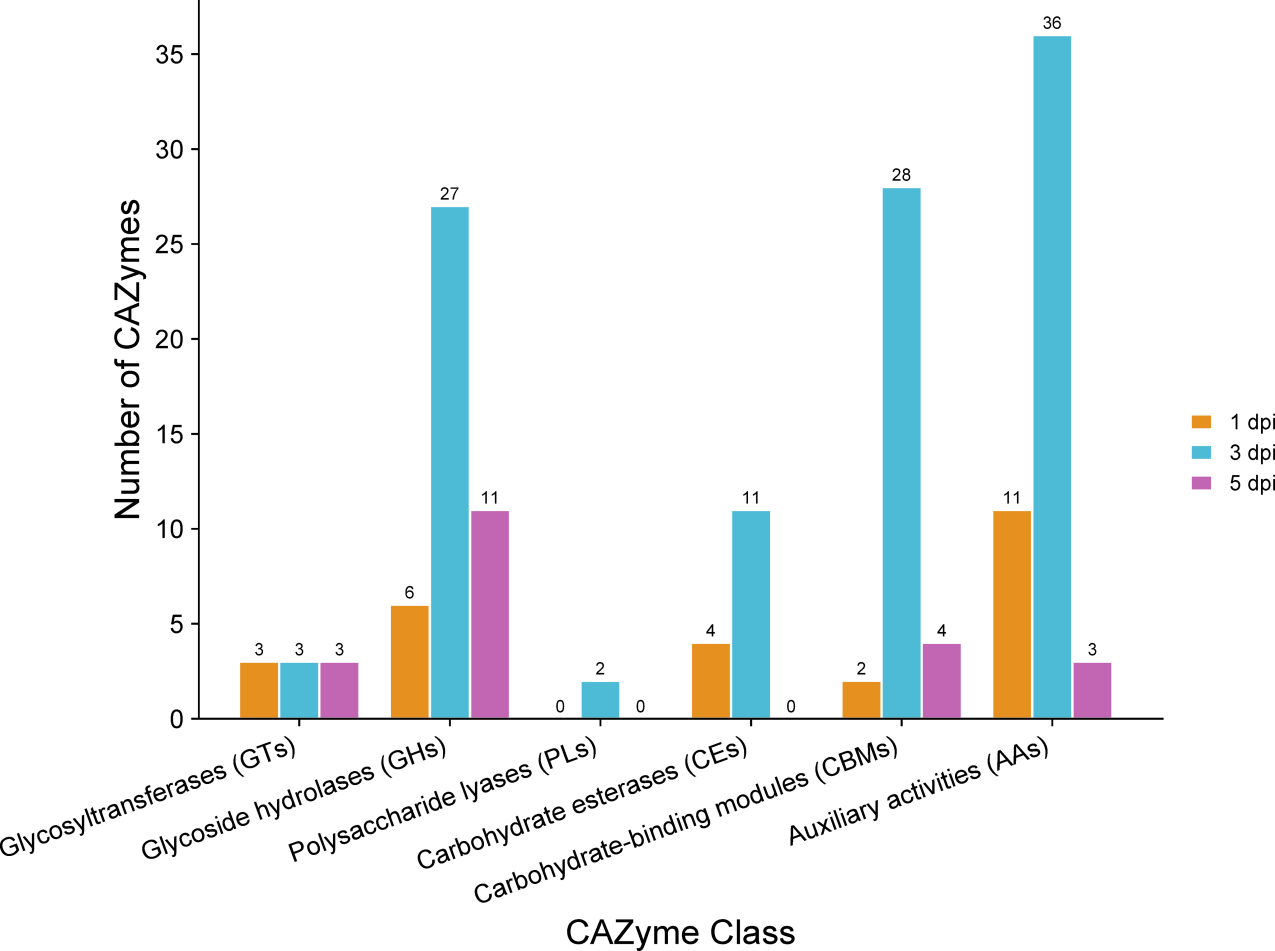
Classes of CAZymes encoded by *C. sublineola* genes up-regulated in the *in planta* versus *in vitro* samples at different time points. Figure created with SRPlot (Tang et al., 2023).

Analysis of proteins encoded by significantly up-regulated genes revealed 17 predicted CAZymes expressed at 1 dpi, 50 at 3 dpi, and 17 at 5 dpi (**Supplementary Data 2**). It is noteworthy that certain genes were predicted to encode enzymes that represent multiple classes of CAZymes (**Supplementary Data 2**). A detailed classification of these CAZymes at each time point highlighted the prevalence of auxiliary activities (AA) (**Figure 8**), referring to proteins defined by their role in assisting other CAZymes in the degradation of the plant cell wall (Levasseur et al., 2013). Specifically, AA enzymes facilitate the breakdown of lignocellulosic biomass by catalyzing redox reactions, often involving the generation of reactive oxygen species (ROS) (Levasseur et al., 2013). This oxidative activity can contribute to the depolymerization of lignin so that the polysaccharides in plant cell walls become better accessible to CAZymes with hydrolytic activity (Levasseur et al., 2013). Indeed, the GHs were prominently represented among the CAZymes. GHs hydrolyze the glycosidic bond between two or more carbohydrates, or between a carbohydrate and a non-carbohydrate moiety, such as a protein or a lipid (Cantarel et al., 2009). GHs are involved in the degradation of cellulose, hemicellulosic polysaccharides, and pectins (Rafiei et al., 2021). The next abundant class of CAZymes encoded by *C. sublineola* genes up-regulated during infection are carbohydrate-binding modules (CBMs; Cantarel et al., 2009). CBMs enhance the activity of enzymes by targeting and promoting a prolonged interaction with the substrate (reviewed by Sidar et al. (2020)) and are most often linked to catalytic modules of other CAZymes present in the same polypeptide. In contrast, PLs were much less abundant. PLs mainly degrade glycosaminoglycans and pectins (Yip and Withers, 2006; Cantarel et al., 2009), which are not major components of the sorghum cell wall.

### 3.5 Predicted fungal effectors secreted by *C. sublineola*

**Figure 9:**
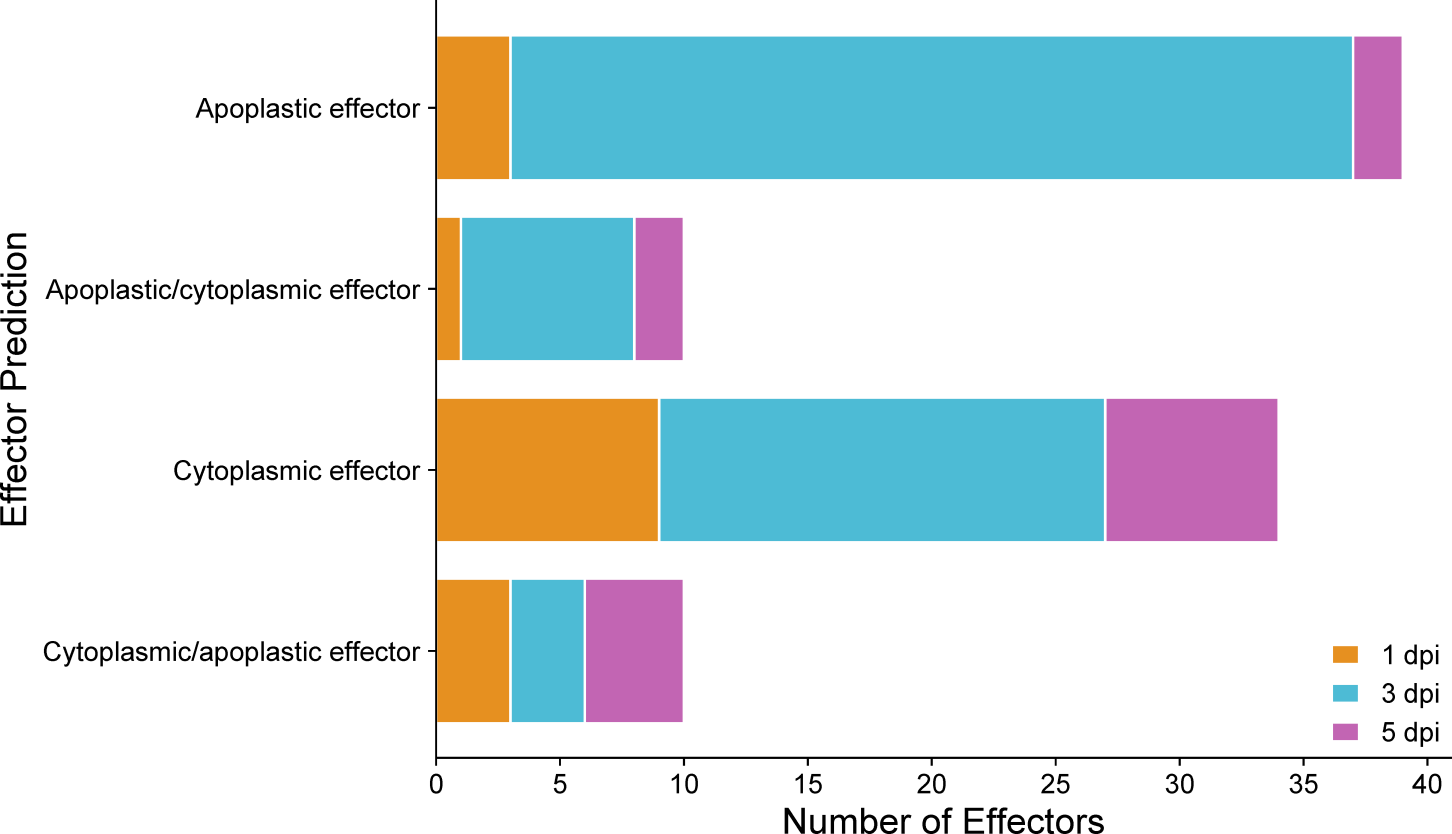
Predicted localization of effectors encoded by *C. sublineola* genes up-regulated in the *in planta* versus *in vitro* samples at different time points. Figure created with SRPlot (Tang et al., 2023). When both the apoplast and cytoplasm are listed as predicted locations, the location listed first has the higher prediction score.

Fungal effectors are characterized as small secreted proteins (SSPs) that primarily act to modulate host physiology, often by suppressing host defenses or shielding the pathogen from the host’s defense responses intended to impede pathogen growth (de Jonge et al., 2011). Some effectors are translocated and function within the host cell’s cytoplasm, while others operate in the extracellular space outside the plant cell (apoplast) (de Jonge et al., 2011). Significantly up-regulated *C. sublineola* genes in the *in planta* versus *in vitro* samples by time point post-inoculation and by accession were analyzed to determine if they encode putative fungal effectors (**Supplementary Data 3**). All predicted effectors expressed by time point are summarized in **Supplementary Data 3**. At 1 dpi, a total of 16 genes encoding predicted effectors were up-regulated. By 3 dpi, this count increased to 62, and at 5 dpi decreased to 15. Several of these genes were expressed at multiple time points (**Supplementary Data 3**). The increase in DEGs encoding predicted effectors at 3 dpi indicated the deployment of effectors facilitating the pathogen’s transition from the biotrophic to the necrotrophic phase. According to the effector localization predictions, most of these candidate effectors are localized in the plant apoplastic space (**Figure 9**; **Supplementary Table 2**). Predicted effectors were compared to proteins in the Pathogen-Host Interaction (PHI) database to infer their roles during different stages of the infection process. A total of 19 predicted *C. sublineola* effectors were highly similar (threshold e-value of 1 × 10^−5^) to known pathogenicity and virulence factors of other fungal pathogens (**Supplementary Data 3**). A number of these were of particular interest because they provide insights into the way *C. sublineola* interacts with its host, as discussed below.

At 1 dpi the *C. sublineola* gene *CSUB01_08309* was up-regulated in the *in planta* versus *in vitro* samples. This gene encodes a predicted effector (A0A066XPB8_COLSU) with a sequence similar to *Sclerotinia sclerotiorum* cutinase 1 (SsCut1; e-value = 8 × 10^−91^) (**Supplementary Data 3**). *S. sclerotiorum* secretes SsCut1 to disrupt the cuticle layer of *Arabidopsis thaliana* and increased expression of *SsCut1* promotes the virulence of *S. sclerotiorum* (Gong et al., 2022). Additionally, at 1 dpi *C. sublineola* gene *CSUB01_05831* is up-regulated. It is predicted to encode a deuterolysin (metalloprotease) protein (A0A066XJ51_COLSU) that has significant similarity (e-value = 1 × 10^−11^) to avirulence (Avr) effector Avr-Pita encoded by *Magnaporthe oryzae* during the infection of rice, causing rice blast disease (**Supplementary Data 3**). Avr-Pita accumulates in the biotrophic interfacial complex (BIC) and is delivered into host cells by invasive hyphae (Khang et al., 2010). Avr-Pita targets the host mitochondria and interacts with the cytochrome *c* oxidase (COX) assembly protein OsCOX11, a key regulator of mitochondrial ROS metabolism in rice (Han et al., 2021). Avr-Pita enhances COX activity and decreases ROS accumulation, and therefore suppresses host innate immunity by disrupting ROS metabolism in the mitochondria (Han et al., 2021).

Four *C. sublineola* genes expressed at 3 dpi (*CSUB01_08906*, *CSUB01_02278*, *CSUB01_09878*, and *CSUB01_09688*) are predicted to encode effectors (A0A066XNR3_COLSU, A0A066X9Z0_COLSU, A0A066XMA2_COLSU, and A0A066XNJ6_COLSU) that are similar to *M. oryzae* cell death-inducing protein 4 (MoCDIP4) (**Supplementary Data 3**). The effector MoCDIP4 is classified as a member of the glycosyl hydrolase family 61 and targets a heat shock-dynamin protein (HSP40-DRP) complex. The targeting leads to the perturbation of mitochondrial dynamics, thereby inhibiting mitochondria-mediated plant immunity such as ROS production and defense-related gene expression (Xu et al., 2020).

The three *C. sublineola* genes *CSUB01_11922*, *CSUB01_04305*, and *CSUB01_10464* expressed at 3 dpi and 5 dpi encode proteins A0A066XAG3_COLSU, A0A066X6X5_COLSU, and A0A066XAZ6_COLSU, respectively, that share significant similarity to a *Cladosporium fulvum* lysin motif effector Ecp6 (**Supplementary Data 3**). Ecp6 is a chitin‐binding effector and has three lysin motif (LysM) domains that sequester chito‐oligosaccharides released from the cell walls of invading hyphae to prevent their recognition by extracellular chitin‐binding tomato immune receptors (Mesarich et al., 2023). This, in turn, prevents the activation of chitin‐triggered immune responses in tomato (Bolton et al., 2008; de Jonge et al., 2010). These three *C. sublineola* genes encode proteins that also share significant sequence similarity to the Secreted LysM Protein 1 (Slp1) expressed by *M. oryzae* in the infection of rice (Mentlak et al., 2012) (**Supplementary Data 3**). Slp1 accumulates at the interface between the fungal cell wall and the rice plasma membrane and sequesters chitin oligosaccharides to prevent PAMP-triggered immunity, including the production of ROS and plant defense gene expression (Mentlak et al., 2012).

Four genes (*CSUB01_02278*, *CSUB01_10115*, *CSUB01_03746*, and *CSUB01_06499*) predicted to encode endo-1,4-β-xylanases (A0A066X9Z0_COLSU, A0A066XFB3_COLSU, A0A066XQM7_COLSU, and A0A066XGR6_COLSU) are expressed at 3 and 5 dpi and have high similarity to *M. oryzae* endo-β-1,4-xylanases MoXYL1A and MoXYL1B (**Supplementary Data 3**). Endo-β-1,4-xylanases are extracellular enzymes responsible for catalyzing the hydrolysis of xylans (Collins et al., 2005), which include glucuronoarabinoxylans, the predominant hemicellulosic polysaccharide in monocot cell walls (Carpita and Gibeaut, 1993). Thus, endo-1,4-β-xylanases play a significant role in fungal penetration and colonization (Beliën et al., 2006; Dornez et al., 2010) and induce necrosis in host tissues (Walton, 1994). MoXYL1A is proposed to enhance the virulence of *M. oryzae* by disrupting the function of the host chloroplast (Shabbir et al., 2022). On the other hand, MoXYL1B does not play a significant role in virulence. It is, however, essential for the proper asexual reproduction of the fungus (Shabbir et al., 2022).

### 3.6 Structural similarity of *C. sublineola* candidate effectors

Given the subcellular localization predictions by WoLF-PSORT, predicted *C. sublineola* effector protein sequences were evaluated for the presence of known nuclear localization sequences (NLS). Five predicted *C. sublineola* effectors with NLSs were identified, suggesting potential nuclear translocation upon secretion into sorghum (**Supplementary Data 4**). To characterize the ‘effector-ome’ of *C. sublineola* and to gain insights into additional candidate effectors encoded by unannotated genes, protein structural clustering was performed on protein structures generated in AlphaFold from coding sequences. FoldSeek analyses revealed structural similarities between *C. sublineola* candidate effectors and those of other *Colletotrichum* species, as well as significant similarities with effectors of other hemibiothrophic fungi (**Supplementary Data 4**), including *Zymoseptoria tritici* and *M. oryzae* (*Pyricularia oryzae*) (Sánchez-Vallet et al., 2015; Fernandez and Orth, 2018). These results suggest potential conserved infection strategies across hosts, which may be due to their hemibiotrophic lifestyle on monocot hosts.

**Figure 10:**
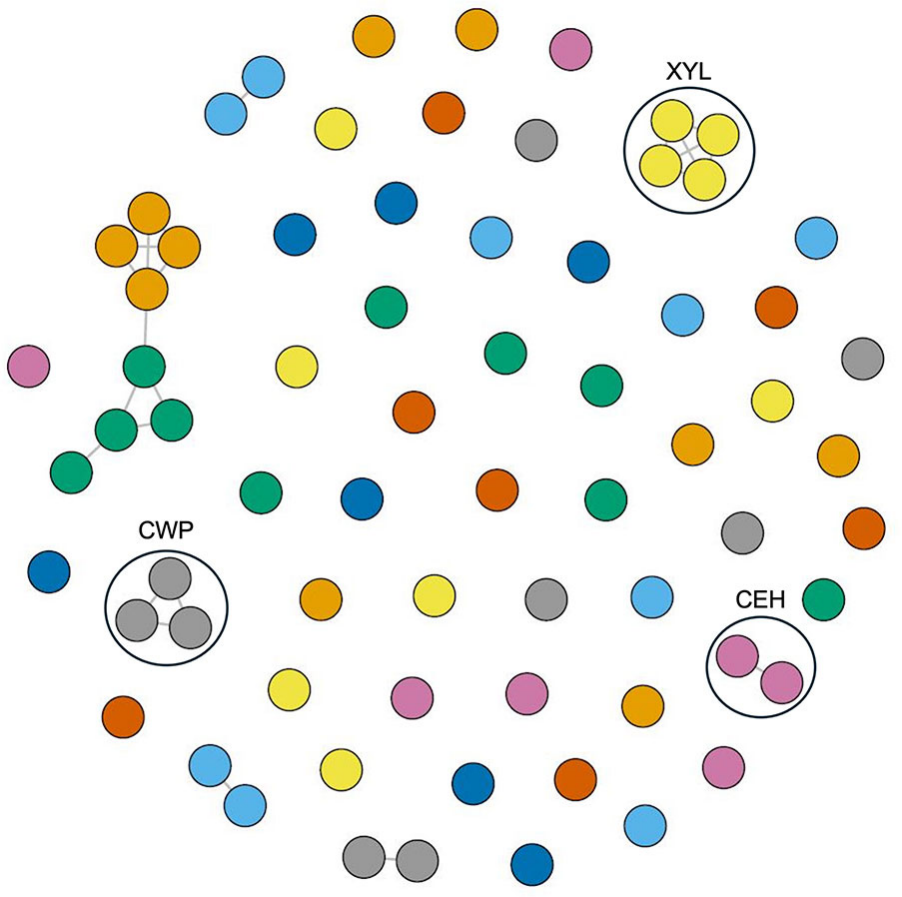
Protein structural network and community analysis of *C. sublineola* effectors. Effectors with a TM-score of 0.6 are considered structurally similar and are connected by edges (lines). Effectors (circles) lacking edges are considered to have no structural similarity to other effectors. Effectors displayed in the same color with edge connections indicate structural similarity. Black circles indicate clusters of effectors with known functions. Abbreviations: Cell wall proteins (CWP), endo-1,4-β-xylanases (XYL), and carboxylic ester hydrolases (CEH).

Additionally, protein structural similarity clustering employing the Louvain community detection method identified eight protein clusters, suggesting distinct functional groups among candidate effectors (**Figure 10**). The majority of the *C. sublineola* candidate effectors did not cluster with other effectors, indicating unique structural features possibly related to specific functions during infection. Notably, three clusters corresponded to cell wall proteins, endo-1,4-β-xylanases, and carboxylic ester hydrolases. These classes of effectors are crucial for the successful infection of *M. oryzae* in rice (Liang et al., 2022; Liu et al., 2022; Lee et al., 2024). The putative effectors A0A066XNJ6_COLSU, A0A066XNR6_COLSU, and A0A066XMA2_COLSU clustered together. Based on PHIB-BLAST analyses, A0A066XNJ6_COLSU and A0A066XMA2_COLSU were predicted to contain a putative fungal cellulose binding domain (also known as carbohydrate binding module), while A0A066XNR6_COLSU may be a member of glycosyl hydrolase family 61. Despite varied annotations, FoldSeek analyses indicated a structural similarity to known endo-β-1,4-glucanase D proteins, implying potentially shared functionalities (**Supplementary Data 4**). Furthermore, the analysis revealed five clusters with at least one uncharacterized protein, that could be indicative of functional similarities based on shared structural features with other members of the cluster. For instance, uncharacterized protein A0A066X3X1_COLSU displayed structural similarity to A0A066X8J3_COLSU, a predicted hydrophobin, which is a member of a class of proteins involved in conidial germination and appressorium development in *M. oryzae* (Kim et al., 2005).

### 3.7 Utilization of membrane transporters by *C. sublineola* to acquire nutrients and mitigate host defense responses

The prolific expression of genes associated with transmembrane transport (**Figure 6**) potentially reflects the different strategies to evade host defenses, deliver effectors, and acquire nutrients from the host. Identified membrane transporters encoded by up-regulated genes were compared to the Pathogen-Host Interaction (PHI) database. Several *C. sublineola* genes expressed at all time points encoded proteins significantly similar to hexose transporters in *Colletotrichum higginsianum* (ChHxt) (**Supplementary Data 5**). A total of six *ChHxt* genes, *ChHxt1* to *ChHxt6*, exhibit specific expression patterns in different infection phases of *C. higginsianum* in *Arabidopsis* (Yuan et al., 2021). *ChHxt4* is required for fungal infection in both biotrophic and necrotrophic stages, while *ChHxt6* is important for the formation of necrotrophic hyphae during infection (Yuan et al., 2021). Thus, the expression of *ChHxt* genes regulates fungal virulence by modulating the utilization of hexoses.

Additionally, several *C. sublineola* genes expressed at all time points encoded proteins significantly similar to ATP-binding cassette (ABC) transporters in various fungal pathogens, most notably the hemibiotroph *M. oryzae* (**Supplementary Data 5**). ABC transporters play pivotal roles in the tolerance and resistance against toxic substances, either by sequestering hydrophobic compounds into specialized organelles or directing them for secretion (Rees et al., 2009; Kim et al., 2013). Investigations into transporter-encoding genes *ABC1* to *ABC4* in *M. oryzae* have revealed their regulatory roles in cytotoxicity, mediation of tolerance against antifungal agents, and resistance to oxidative stress, facilitating the pathogen’s ability for successful infections (Urban, 1999; Lee et al., 2005; Sun et al., 2007; Gupta and Chattoo, 2008). Specifically, *M. oryzae* ABC1 (Urban, 1999) and ABC4 (Gupta and Chattoo, 2008) are crucial for pathogenicity, aiding the fungus in navigating the cytotoxic environment during infection, whereas ABC2 (Lee et al., 2005) and ABC3 contribute to multidrug resistance, with ABC3 playing a specific role in overcoming cytotoxicity and oxidative stress within appressoria during early infection-related morphogenesis (Sun et al., 2007).

### 3.8 Comparison of *C. sublineola* gene expression in host genotypes SC110 and SC1033

We further analyzed genes differentially expressed in the pathogen in sorghum accessions SC110 versus SC1033. The host resistance response in SC110 has been elucidated and involves an increase in the production of reactive oxygen species (Wolf et al., 2024). SC1033 lacks resistance alleles at the three anthracnose resistance loci identified on chromosome 5 (Cuevas et al., 2018). A total of 99 genes were up-regulated and four were down-regulated in *C. sublineola* during infection of SC110 compared to SC1033. Up-regulated genes were compared to the PHI database to determine the known virulence factors implemented by *C. sublineola* to combat the defense response in SC110. A total of 33 up-regulated genes had significant hits (threshold e-value of 1 × 10^−5^) to the PHI database (**Supplementary Data 6**). Up-regulated pathogen genes in SC110 with a substantial number of hits to the PHI database (>50) included the following:

*CSUB01_04556* encodes a calcium-transporting ATPase (A0A066X398_COLSU) with similarity to six calcium pumps (Eca1, Spf1, PmcA/B/C, and Pmcr1) in *Beauveria bassiana*, a fungal pathogen of insects (**Supplementary Data 6**). All six calcium pumps play vital roles in sustaining antioxidant activity and cell wall integrity of *B. bassiana*. Deletion of the genes encoding the *B. bassiana* calcium pumps resulted in increased sensitivities to the oxidants menadione and H_2_O_2_ and cell-wall-perturbing agents, reduced activities of intracellular superoxide dismutases (SODs) and catalases, and altered cell wall components (Wang et al., 2013, 2017).

*CSUB01_10385* encodes a putative ketoreductase (KR) domain-containing protein (A0A066XUL6_COLSU) that displays similarity to several polyketide synthases (PKSs) in a variety of different fungal pathogens (**Supplementary Data 6**). PKSs are enzymes involved in the biosynthesis of polyketides, a diverse class of secondary metabolites contributing to pathogen virulence, host interaction, and environmental adaptation (Fujii, 2010). A0A066XUL6_COLSU is most similar (e-value = 0) to a PKS encoded by *fumonisin biosynthetic gene 1* (*FUM1*) in *Gibberella moniliformis* (**Supplementary Data 6**). *FUM1* encodes an enzyme that catalyzes the synthesis of a polyketide that forms a key constituent of fumonisin (Procter et al., 2003). Fumonisin is a mycotoxin that is known to be a strong inducer of programmed cell death (PCD) in plants (Lanubile et al., 2022). Since PKS-related genes tend to be organized in biosynthetic gene clusters (BGCs), the genomic region near *CSUB01_10385* was analyzed using antiSMASH (Blin et al., 2023) to gain insights into its role during infection. The 20-kb genomic region near *CSUB01_10385* has similarities to several BGCs responsible for the production of toxins in different fungi (**Supplementary Figure 4**). Similarity of BGCs is defined by sequence similarity, gene order/organization, gene composition, regulatory elements, and biological activity. This genomic region in *C. sublineola* shared the most similarity (21%) to a BGC in *Cryphonectria parasitica*, which causes chestnut blight disease, and 9% similarity to a BGC in *Fusarium fujikuroi*. Thus, the BGC identified in *C. sublineola* could function in the production of a pathogen-specific mycotoxin.

*CSUB01_02347* encodes a mitogen-activated protein kinase kinase kinase (MAPKKK) (A0A066X7D3_COLSU) that is most similar (e-value = 0) to MAPKKK Ssk2/Ssk22 in *Fusarium graminearum* (**Supplementary Data 6**). Ssk2/Ssk22 is a component of the high osmolarity glycerol (HOG) mitogen-activated protein kinase (MAPK) signaling pathway, a key element that controls adaptation to environmental stress. The HOG pathway is required for fungal growth under hyperosmotic conditions (Ma and Li, 2013). However, since the MAPK pathway is known to be involved in response to oxidative stress in several fungi (Furukawa et al., 2005; Segmüller et al., 2007), *Fgssk2* mutants are not only hypersensitive to osmotic stress but also have increased sensitivity to oxidative stresses and to chemicals that mimic cytoplasmic membrane and cell wall stresses (Zheng et al., 2012).

### 3.9 Assessing pathogenicity of *C. sublineola* using aniline blue staining and fluorescence microscopy

**Figure 11:**
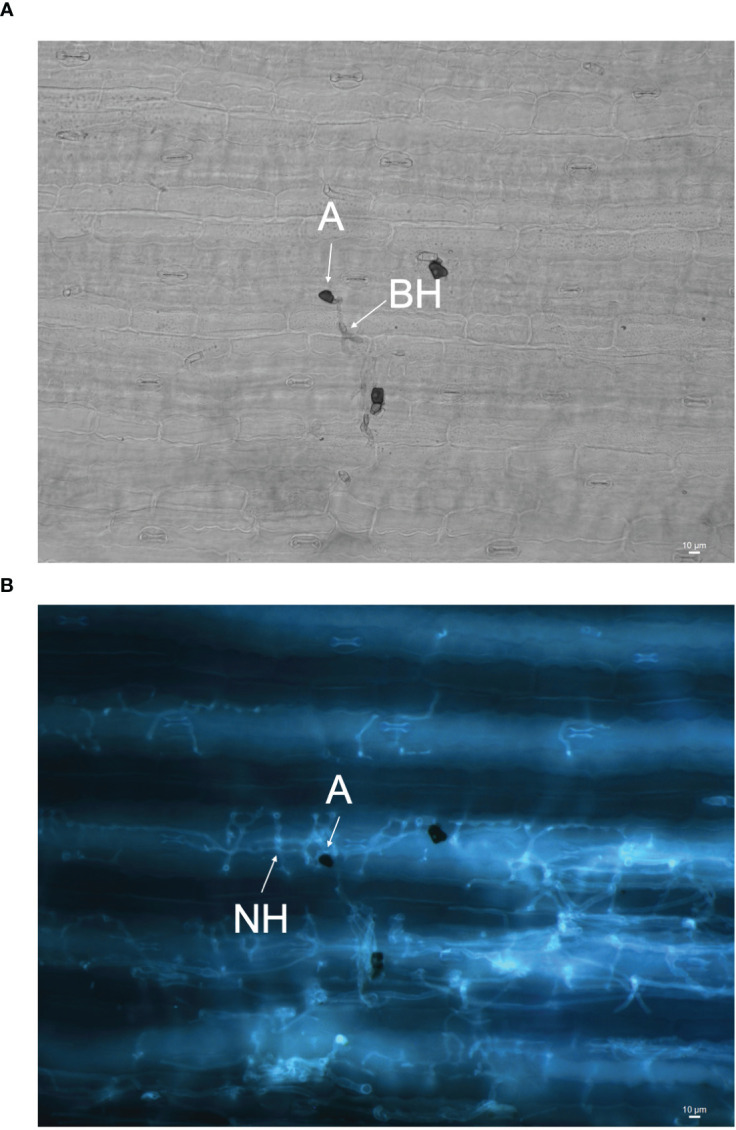
Images of *C. sublineola* infecting genotype SC1033 at 5 dpi. **(A)** A melanized appressorium (A) gives rise to biotrophic hyphae (BH) visible with light microscopy in this adaxial view of cleared tissue. **(B)** Necrotrophic hyphae (NH) associated with the same appressorium are visible using fluorescence microscopy following staining with aniline blue. Images captured at 20× magnification. Scale bars indicate 10 μm.

**Figure 12:**
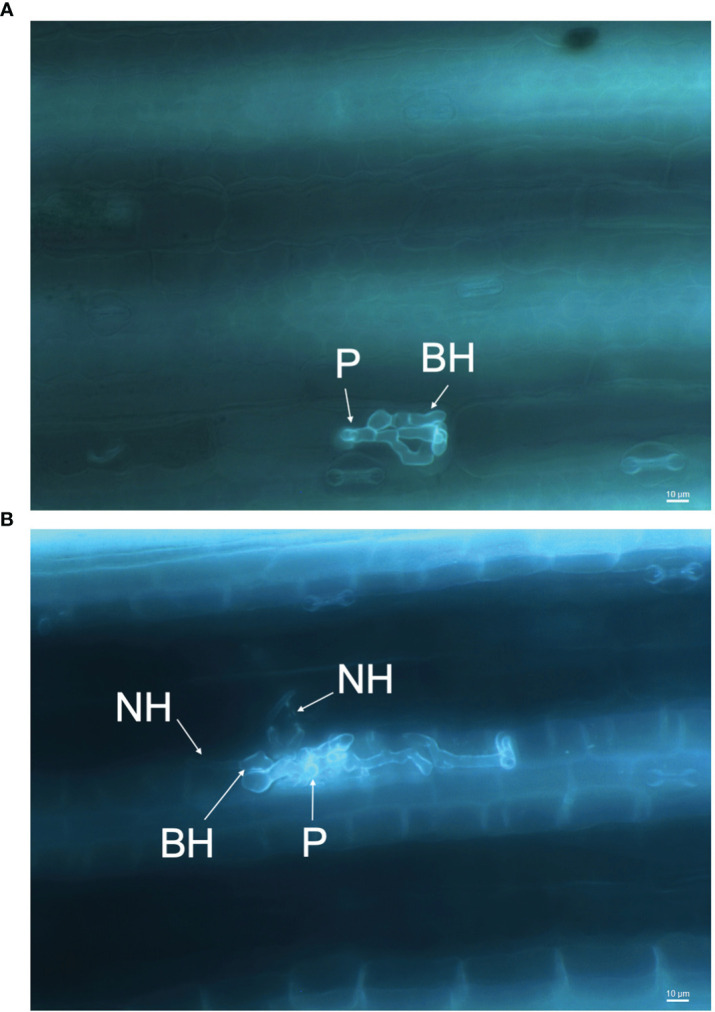
Infection process of *C. sublineola* on sorghum genotype SC1033. Images captured at 40× magnification of the adaxial side of the leaf following the clearing of pigments and staining with aniline blue. **(A)** Between 1 and 3 days post-infection (dpi) the appressorium forms an infection peg (P) allowing penetration of the host cuticle and epidermal cell wall. After penetration, an infection vesicle is formed in the host epidermal cell and develops biotrophic hyphae (BH). **(B)** Between 3 and 5 dpi, BH grow intracellularly, colonizing several host cells, and begin to give rise to distinctly thinner necrotrophic hyphae (NH). Scale bars indicate 10 μm.

The infection processes of *C. sublineola* in sorghum genotype SC1033 were observed with epi-fluorescence and light microscopy. The lunate conidia were not visible with aniline blue staining at any of the selected time points. However, Wharton et al. (2001) reported conidia to produce germ tubes that form globose melanized appressoria. Therefore, conidia are located in close proximity to the appressoria. Appressoria were visible via light microscopy at 3 and 5 dpi (**Figure 11A**). Once formed, the appressorium punctures the host cell wall visible by a penetration pore in which the infection peg emerges (**Figure 12**). At 3 dpi, *C. sublineola* developed bulbous, intracellular, biotrophic hyphae (**Figure 12A**). After 3 dpi, biotrophic hyphae grew intracellularly, colonizing several host epidermal cells (**Figure 12B**). The intracellular growth of the biotrophic hyphae was characterized by the constriction of hyphae between cells consistent with observations by Wharton et al. (2001). Eventually, biotrophic hyphae gave rise to thin necrotrophic hyphae (**Figure 12B**). By 5 dpi, necrotrophic hyphae displayed intracellular, intercellular, and subcuticular growth throughout the mesophyll and vascular tissue (**Figure 11B**).

## 4 Discussion

The lifestyle patterns of *Colletotrichum* species are highly regulated by specific gene families that play pivotal roles in orchestrating the pathogen’s response to host defenses, nutrient acquisition, and environmental cues. For instance, transcriptome analyses of *C. higginsianum* infecting *Arabidopsis thaliana* and *C. graminicola* infecting maize revealed that different categories of genes are transcribed in successive waves that are linked to pathogenic transitions: effectors and secondary metabolism enzymes are induced before penetration and during biotrophy, whereas most hydrolases and transporters are produced later, at the switch to necrotrophy (O’Connell et al., 2012). Conversely, in *C. sublineola* we observed that all main gene categories increased in expression during the early transition from the biotrophic phase to the necrotrophic phase (3 dpi). At this stage, prominent gene categories relevant to *C. sublineola*’s pathogenicity were carbohydrate metabolism and transmembrane transport (**Figure 6**). Therefore, subsequent analysis focused on the identification of virulence factors that could be classified in these categories.

Comparative genome analyses across the genus have revealed that *Colletotrichum* species exhibit the presence/absence of genes encoding carbohydrate-degrading enzymes tailored to their specific lifestyles and hosts (Gan et al., 2013). *C. sublineola* deploys a repertoire of CAZymes that reflect the composition of the host cell wall. Sorghum cell walls are comprised mainly of cellulose (45%), hemicellulosic polysaccharides (20–25%), lignin (18–22%), and pectin (3–5%) (Castro et al., 2017). Thus, cellulases, hemicellulases, and enzymes that degrade lignocellulosic structures represented the majority of CWDEs. In contrast, there was a limited upregulation of genes encoding pectinases. This result is consistent with reports in other *Colletorichum* species. *C. higginsianum*, which infects Arabidopsis, possesses more than twice the number of pectin-degrading enzymes compared to *C. graminicola*, a maize pathogen (O’Connell et al., 2012). This difference mirrors their host preferences: the primary cell walls of dicots such as Arabidopsis contain 20–35% pectin versus 5% in the primary cell walls of grasses (Carpita and Gibeaut, 1993; Vogel, 2008; O’Connell et al., 2012).

**Figure 13:**
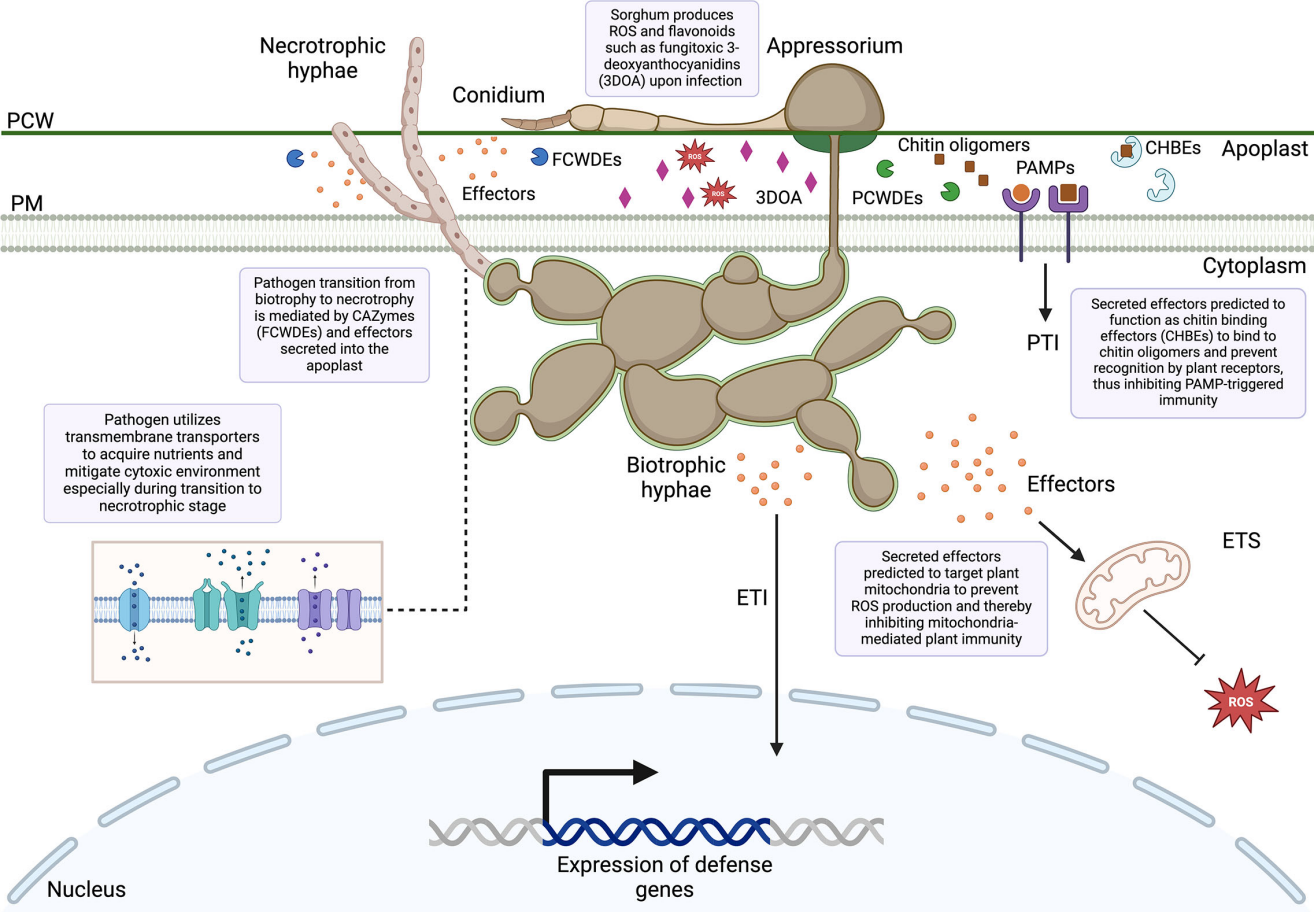
Overview of sorghum–*Colletotrichum sublineola* interactions uncovered from the dual RNA-seq. PCW, Plant cell wall; PM, plasma membrane; FCWDEs, fungal cell wall degrading enzymes; PCWDEs, plant cell wall degrading enzymes; PAMPs, pathogen-associated molecular patterns; PTI, PAMP-triggered immunity; 3DOA, 3-deoxyanthocyanidins; CHBEs, chitin-binding effectors; ROS, reactive oxygen species; ETI, effector-triggered immunity; ETS, effector-triggered susceptibility.

Apoplastic effectors interfere with PAMP perception mediated by plant membrane-bound pattern recognition receptors (PPR), leading to the inactivation of NADPH oxidases (Jwa and Hwang, 2017). By contrast, cytoplasmic effectors target the MAPK signaling pathway, vesicle trafficking, and metabolic priming, which are essential for apoplastic ROS production (Jwa and Hwang, 2017). The majority of predicted effectors secreted by *C. sublineola* were localized in the apoplast (**Figure 9**), indicating that *C. sublineola* targets the PAMP-immunity pathway likely during the initial colonization of the apoplastic space by necrotrophic hyphae (**Figures 12B**, **13**). During the transition from biotrophy to necrotrophy, *C. sublineola* expressed chitin‐binding effectors that sequester chito‐oligosaccharides released from the cell walls of invading hyphae to prevent their recognition by extracellular chitin‐binding immune receptors, thus preventing the activation of PAMP-triggered immunity (**Figure 13**). Previous expression analyses have identified *chitinase* (*PR3*) and *β-1,3-glucanase* to be pathogen-inducible genes in sorghum, indicating that the *C. sublineola* cell wall serves as a PAMP that is detected by the host receptors (Li et al., 2013; Ahn et al., 2019). Our expression data suggest that *C. sublineola* secretes specific effectors in the apoplast to prevent recognition via PTI.

It has been well documented that inducible defense responses in resistant genotypes of sorghum include early accumulation of ROS, such as hydrogen peroxide (H_2_O_2_), and phytoalexins, which decrease the formation of appressoria (Basavaraju et al., 2009). ROS act as cellular signaling molecules to trigger plant immune responses. We uncovered candidate effectors that target multiple steps in the ROS signaling pathway in the host (**Figure 13**). Several identified candidate effectors are predicted to target mitochondria, where they may alter mitochondrial morphology and metabolism. Mitochondria play an active role in altering intracellular metabolism to defend against pathogen attack, mediating hormone-driven signaling, and facilitating signal transduction. This involvement leads to the generation of ROS and reactive nitrogen species, ultimately triggering programmed cell death (Xu et al., 2020). Moreover, mitochondria function as a signaling platform in mammals, activating downstream immune responses upon the recognition of viral pathogen-associated molecular patterns (PAMPs) (Angajala et al., 2018; Mohanty et al., 2019). Similarly, in plant cells, mitochondria could serve as a signaling organelle, amplifying defense responses through the activation of various signals such as nitric oxide, ROS, or salicylic acid (Colombatti et al., 2014).

The phenylpropanoid and flavonoid pathways are the central hub of the metabolism producing anti-fungal compounds in response to anthracnose infection in sorghum (Li et al., 2013; Tugizimana et al., 2019). Phytoalexins produced uniquely by sorghum include a group of flavonoids known as 3-deoxyanthocyanidins, primarily luteolinidin and apigeninidin (Huang and Backhouse, 2004). Notably, at 1 dpi the host flavonoid biosynthesis-related genes are down-regulated (**Figure 4A**) while they are highly up-regulated at 3 dpi (**Figure 4B**) and 5 dpi (**Figure 4C**). This observation indicates that *C. sublineola* could evade early (<3 dpi) host defense responses by compromising flavonoid production with the help of secreted effectors. Manipulation of flavonoid metabolism has been reported for the biotrophic fungus *Ustilago maydis* (now called *Mycosarcoma maydis*), the causal agent of smut in maize, in which the virulence-promoting secreted effector protein Tin2 is responsible for diverting intermediates used for the defense-related production of lignin by inducing anthocyanin biosynthesis (Tanaka et al., 2014).

Previous studies have demonstrated that early flavonoid phytoalexin accumulation in sorghum is important in preventing the proliferation of *C. sublineola* fungal hyphae during infection (Wharton et al., 2001; Basavaraju et al., 2009). The vesicles surrounding the infection site accumulate 3-deoxyanthocyanidins, and associate with the plasma membrane. This process results in the host cell collapsing and the release of fungitoxic 3-deoxyanthocyanidins into both the apoplast and the pathogen (Nielsen et al., 2005; Buiate et al., 2017). 

Genes encoding transmembrane transporters were expressed across all time points by *C. sublineola*, similar to what has been observed for *M. oryzae*, indicating a potential strategy for *C. sublineola* to overcome sorghum’s early defense responses by mitigating the effects of cytotoxic compounds and oxidative stress (**Figure 13**) (Urban, 1999; Lee et al., 2005; Sun et al., 2007; Gupta and Chattoo, 2008).

Since many genes similar to *ChHxt* hexose transporters are expressed throughout infection, it suggests a diversity of transporters is required to allocate nutrients from the changing environment in the host during the switch from the biotrophic phase to the necrotrophic phase. The necrotrophic infection phase occurs when secondary (necrotrophic) hyphae, which are distinctly thinner than primary (biotrophic) hyphae, begin to spread throughout host tissue. Necrotrophy begins at approximately 3 dpi (**Figure 12B**). Based on our analyses, the transition between biotrophy and necrotrophy is facilitated by CAZymes, transmembrane transporters, and secreted effectors. Notably, we uncovered genes encoding biotrophy-associated secreted protein 3 (BAS3) as a predicted secreted effector at 3 dpi (**Supplementary Data 2**), which is known to facilitate this transition in *M. oryzae*, via secretion by invasive hyphae (Wang et al., 2019). During this transition, it is characteristic of *Colletotrichum* species to produce toxins that kill host tissue, which rapidly becomes necrotic (Münch et al., 2008). Although toxin biosynthetic genes did not represent a main fungal gene category during infection, an up-regulation of genes associated with potential toxin biosynthetic genes (**Supplementary Figure 4**) is observed at 3 and 5 dpi (**Figure 6**), further indicating the switch to necrotrophy occurs within this timeframe.

Wolf et al. (2024) proposed that the F-box protein encoded by *Sobic.005G172300* targets proteins involved in the biosynthesis of ascorbic acid for polyubiquitination through the SCF E3 ubiquitin ligase, causing their degradation via the proteasome. Hence, the upregulation of *Sobic.005G172300* in SC110 results in the transcriptional downregulation of key genes encoding ascorbic acid biosynthetic enzymes. In turn, the decreased concentration of ascorbic acid generates an increase in ROS. Accumulation of ROS by the host damages fungal cells and hinders the pathogen’s ability to establish infection. Here, we uncovered several *C. sublineola* genes up-regulated during infection of SC110 are associated with mitigating oxidative stress as well as genes necessary for fungal cell wall integrity likely to combat the degradation of the fungal cell wall due to host ROS. Moreover, genes responsible for the biosynthesis of putative toxins were up-regulated. Oxidative stress is often a prerequisite for toxin production (Reverberi et al., 2005, 2010). ROS can stimulate the expression of genes involved in toxin biosynthesis in fungi by acting as signaling molecules. For example, the cereal pathogen *F. graminearum* produces the mycotoxin deoxynivalenol (DON), which is a virulence factor induced by ROS (Nguyen et al., 2013). Therefore, the accumulation of host ROS in SC110 may trigger the production of toxins by *C. sublineola* to enhance pathogen defense in response to the oxidative burst.

The dual RNA-sequencing approach presented here improves our current knowledge of the molecular mechanisms between the sorghum and *C. sublineola* interaction (**Figure 13**). Notably, the identification of secreted predicted effectors provides valuable insights into the functional diversity and potential roles of these proteins during infection. These data provide a foundation for subsequent experimental validation, such as the transient expression of fluorescent protein-tagged effectors *in planta* that could confirm their contributions to pathogen virulence. Given the genetic diversity of *C. sublineola*, experiments conducted with isolates from diverse geographic locations in which the -omics approaches used in this study are combined with high-throughput genomics tools (*e.g*., genotyping by sequencing) will form the basis for the effective protection of sorghum against this aggressive fungal pathogen.

## Data availability statement

The original contributions presented in the study are publicly available. The RNA-sequencing data are deposited at the National Center for Biotechnology Information (NCBI) Sequence Read Archive (SRA). The BioProject ID for the data representing the sorghum samples is PRJNA961726, and for the data from the *in vitro* cultured *C. sublineola* is PRJNA1114779.

## Author contributions

SV: Conceptualization, Data curation, Formal analysis, Investigation, Methodology, Visualization, Writing – original draft, Writing – review & editing. EW: Investigation, Methodology, Writing – review & editing, Writing – original draft. JR: Conceptualization, Investigation, Methodology, Resources, Supervision, Writing – review & editing. HC: Conceptualization, Funding acquisition, Methodology, Writing – review & editing. WV: Conceptualization, Funding acquisition, Investigation, Project administration, Resources, Supervision, Writing – review & editing.
