# Supplementary material for: Dual-RNA-sequencing to elucidate the interactions between sorghum and Colletotrichum sublineola
Source: Front Fungal Biol. 2024 Aug 16;5:1437344. doi: 10.3389/ffunb.2024.1437344 (PMC11362643; doi:10.3389/ffunb.2024.1437344)
Supplement: Supplementary file 2 [file Datasheet2.docx]

Supplementary Material

# Supplementary Data

**Data file 1** RNA-mapping statistics and sequencing quality.

**Data file 2** CAZyme prediction and classifications.

**Data file 3** Predicted effectors, effector localization, and PHIB-BLAST results.

**Data file 4** Effector clustering matrices and results.

**Data file 5** Predicted transmembrane transporters and PHIB-BLAST results.

**Data file 6** Differentially expressed genes by *C. sublineola* in sorghum genotypes SC110 versus SC1033 PHIB-BLAST results.

# Supplementary Tables

**Supplementary Table 1.** Resistant and susceptible refers to the presence or absence of resistance alleles, respectively, at the corresponding locus in each genotype (Cuevas et al., 2018; Wolf et al., 2024).

| Genotype | ID | *Sobic.05G172300* | *Sobic.05G182400* | *Sobic.05G228400* |
| --- | --- | --- | --- | --- |
| SC17 | PI 533903 | Susceptible | Susceptible | Resistant |
| SC110 | PI 533794 | Resistant | Susceptible | Susceptible |
| SC1033 | PI 576426 | Susceptible | Susceptible | Susceptible |
| SC1330 | PI 597973 | Susceptible | Resistant | Susceptible |

**Supplementary Table 2.** Fungal effectors predicted using the pipeline of Lu et al. (2022) based on up-regulated genes *in planta* versus *in vitro* samples by days post-infection (dpi). The table includes the *C. sublineola* protein identifier retrieved from UniProt, the annotation, the predicted location of the effector, the time point(s) at which the effector was upregulated, and hits to the Pathogen Host Interaction database using PHIB-BLAST (Urban et al., 2019). Some effectors have two localization predictions in which the location listed first has the higher prediction score.

| Identifier | | Annotation | Localization prediction | Time point(s) expressed | PHIB-BLAST Hit (e-value≤10^-5^) | |
| --- | --- | --- | --- | --- | --- | --- |
| A0A066X8X4_COLSU | | Uncharacterized protein | Cytoplasmic/apoplastic | 1,3,5 |  | |
| A0A066XC28_COLSU | | Small secreted protein | Cytoplasmic | 1,3,5 |  | |
| A0A066XIB3_COLSU | | CsbD-like domain-containing protein | Cytoplasmic | 1,3,5 |  | |
| A0A066XCL3_COLSU | | Secreted protein | Cytoplasmic/apoplastic | 1,3,5 |  | |
| A0A066XD31_COLSU | | Putative CFEM domain-containing protein | Apoplastic/cytoplasmic | 1,3,5 |  | |
| A0A066XW89_COLSU | | Uncharacterized protein | Cytoplasmic | 1,3,5 |  | |
| A0A066XBV1_COLSU | | Pectin esterase | Apoplastic | 1,3 | Y | |
| A0A066XGG6_COLSU | | Uncharacterized protein | Cytoplasmic | 1,3 |  | |
| A0A066XIY2_COLSU | | Uncharacterized protein | Cytoplasmic | 1,3 |  | |
| A0A066XH10_COLSU | | Cell wall protein | Cytoplasmic | 1,3 | Y | |
| A0A066XJL9_COLSU | | Cell wall protein | Cytoplasmic/apoplastic | 1,5 |  | |
| A0A066XLV3_COLSU | | Small secreted protein | Cytoplasmic | 1,5 |  | |
| A0A066XLM2_COLSU | | Uncharacterized protein | Cytoplasmic | 1,5 |  | |
| Identifier | Annotation | Localization prediction | Time point(s) expressed | PHIB-BLAST Hit (e-value≤10^-5^) |  |  |
| A0A066XZD4_COLSU | | Uncharacterized protein | Cytoplasmic | 3,5 |  | |
| A0A066XAG3_COLSU | | Putative intracellular hyphae protein | Apoplastic | 3,5 | Y | |
| A0A066XF37_COLSU | | Cell wall protein | Apoplastic | 3,5 | Y | |
| A0A066X6X5_COLSU | | Putative LysM domain-containing protein | Apoplastic | 3,5 | Y | |
| A0A066XRT8_COLSU | | Uncharacterized protein | Cytoplasmic | 3,5 |  | |
| A0A066XPB8_COLSU | | Putative cutinase | Apoplastic | 1 | Y | |
| A0A066XN81_COLSU | | Putative glycosyl hydrolase | Apoplastic | 1 | Y | |
| A0A066X254_COLSU | | Uncharacterized protein | Cytoplasmic | 1 |  | |
| A0A066WV46_COLSU | | Uncharacterized protein | Cytoplasmic | 3 |  | |
| A0A066XJ51_COLSU | | Deuterolysin | Apoplastic | 3 | Y | |
| A0A066XWK9_COLSU | | Uncharacterized protein | Apoplastic | 3 |  | |
| A0A066WY31_COLSU | | Putative peptidase A4 family | Apoplastic | 3 |  | |
| A0A066X848_COLSU | | Biotrophy-associated secreted protein 3 | Apoplastic | 3 |  | |
| A0A066X0J9_COLSU | | Carboxylic ester hydrolase | Apoplastic | 3 |  | |
| A0A066XLS1_COLSU | | Uncharacterized protein | Apoplastic | 3 |  | |
| A0A066XCN6_COLSU | | Uncharacterized protein | Apoplastic | 3 |  | |
| Identifier | Annotation | Localization prediction | Time point(s) expressed | PHIB-BLAST Hit (e-value≤10^-5^) |  |  |
| A0A066X3X1_COLSU | Uncharacterized protein | Apoplastic | 3 |  |  |  |
| A0A066XRP4_COLSU | Putative glycoside hydrolase family 16 | Apoplastic | 3 |  |  |  |
| A0A066X9Z0_COLSU | Endo-1,4-beta-xylanase | Apoplastic | 3 | Y |  |  |
| A0A066XJW7_COLSU | Uncharacterized protein | Apoplastic/cytoplasmic | 3 |  |  |  |
| A0A066XMA2_COLSU | Putative fungal cellulose binding domain-containing protein | Apoplastic | 3 | Y |  |  |
| A0A066X6U8_COLSU | Celp0028 effector like protein | Apoplastic | 3 |  |  |  |
| A0A066X6X5_COLSU | Putative LysM domain-containing protein | Apoplastic | 3,5 | Y |  |  |
| A0A066XRT8_COLSU | Uncharacterized protein | Cytoplasmic | 3,5 |  |  |  |
| A0A066XPB8_COLSU | Putative cutinase | Apoplastic | 1 | Y |  |  |
| A0A066XN81_COLSU | Putative glycosyl hydrolase | Apoplastic | 1 | Y |  |  |
| A0A066X254_COLSU | Uncharacterized protein | Cytoplasmic | 1 |  |  |  |
| A0A066WV46_COLSU | Uncharacterized protein | Cytoplasmic | 3 |  |  |  |
| A0A066XJ51_COLSU | Deuterolysin | Apoplastic | 3 | Y |  |  |
| A0A066XWK9_COLSU | Uncharacterized protein | Apoplastic | 3 |  |  |  |
| A0A066WY31_COLSU | Peptidase A4 family | Apoplastic | 3 |  |  |  |
| Identifier | Annotation | Localization prediction | Time point(s) expressed | PHIB-BLAST Hit (e-value≤10^-5^) |  |  |
| A0A066X848_COLSU | Biotrophy-associated secreted protein 3 | Apoplastic | 3 |  |  |  |
| A0A066X0J9_COLSU | Carboxylic ester hydrolase (Fragment) | Apoplastic | 3 |  |  |  |
| A0A066XLS1_COLSU | Uncharacterized protein | Apoplastic | 3 |  |  |  |
| A0A066XCN6_COLSU | Uncharacterized protein | Apoplastic | 3 |  |  |  |
| A0A066X3X1_COLSU | Uncharacterized protein | Apoplastic | 3 |  |  |  |
| A0A066XRP4_COLSU | Putative glycoside hydrolase family 16 | Apoplastic | 3 |  |  |  |
| A0A066X9Z0_COLSU | Endo-1,4-beta-xylanase | Apoplastic | 3 | Y |  |  |
| A0A066XJW7_COLSU | Uncharacterized protein | Apoplastic/cytoplasmic | 3 |  |  |  |
| A0A066XMA2_COLSU | Putative fungal cellulose binding domain-containing protein | Apoplastic | 3 | Y |  |  |
| A0A066X6U8_COLSU | Celp0028 effector like protein | Apoplastic | 3 |  |  |  |
| A0A066XNJ6_COLSU | Putative glycosyl hydrolase family 61 | Apoplastic | 3 | Y |  |  |
| A0A066XXE9_COLSU | Uncharacterized protein | Apoplastic | 3 |  |  |  |
| A0A066WZI1_COLSU | Uncharacterized protein | Cytoplasmic | 3 |  |  |  |
| A0A066XFL6_COLSU | Uncharacterized protein | Apoplastic | 3 |  |  |  |
| A0A066XJ45_COLSU | Carboxylic ester hydrolase | Apoplastic | 3 | Y |  |  |
| Identifier | Annotation | Localization prediction | Time point(s) expressed | PHIB-BLAST Hit (e-value≤10^-5^) |  |  |
| A0A066WZI1_COLSU | Uncharacterized protein | Cytoplasmic | 3 |  |  |  |
| A0A066XFL6_COLSU | Uncharacterized protein | Apoplastic | 3 |  |  |  |
| A0A066XJ45_COLSU | Carboxylic ester hydrolase | Apoplastic | 3 | Y |  |  |
| A0A066WXB0_COLSU | Uncharacterized protein | Cytoplasmic | 3 |  |  |  |
| A0A066X3D8_COLSU | Cyanovirin-N domain-containingprotein | Apoplastic | 3 |  |  |  |
| A0A066X8J3_COLSU | Hydrophobin | Apoplastic | 3 |  |  |  |
| A0A066XUW8_COLSU | Uncharacterized protein | Apoplastic | 3 |  |  |  |
| A0A066XAD1_COLSU | Uncharacterized protein | Apoplastic | 3 |  |  |  |
| A0A066XQM7_COLSU | Endo-1,4-beta-xylanase | Apoplastic | 3 | Y |  |  |
| A0A066XRK6_COLSU | Uncharacterized protein | Cytoplasmic | 3 |  |  |  |
| A0A066X2J5_COLSU | Putative cholera enterotoxin A2 | Cytoplasmic | 3 |  |  |  |
| A0A066XAZ6_COLSU | Putative LysM protein | Apoplastic | 3 | Y |  |  |
| A0A066XTQ1_COLSU | Cytochrome P450 | Apoplastic | 3 |  |  |  |
| A0A066XGR6_COLSU | Endo-1,4-beta-xylanase | Apoplastic/cytoplasmic | 5 | Y |  |  |

#

# Supplementary Figures

#

**Supplementary Figure 1.** Progression of disease symptoms on leaves of sorghum accessions SC17, SC110, SC1033, and SC1330 at 1, 3, and 5 days post-infection (dpi) with *C. sublineola*. Plants at the fifth-leaf stage were spray-inoculated with a conidial suspension (1×10^6^ condida/mL).

**Supplementary Figure 2.** Principal component score plot of expression profiles of *C. sublineola* *in planta* (inoculated) samples across sorghum genotypes. Inoculated samples were collected at 1 dpi (yellow), 3 dpi (pink), and 5 dpi (blue). Figures created in OmicsBox.


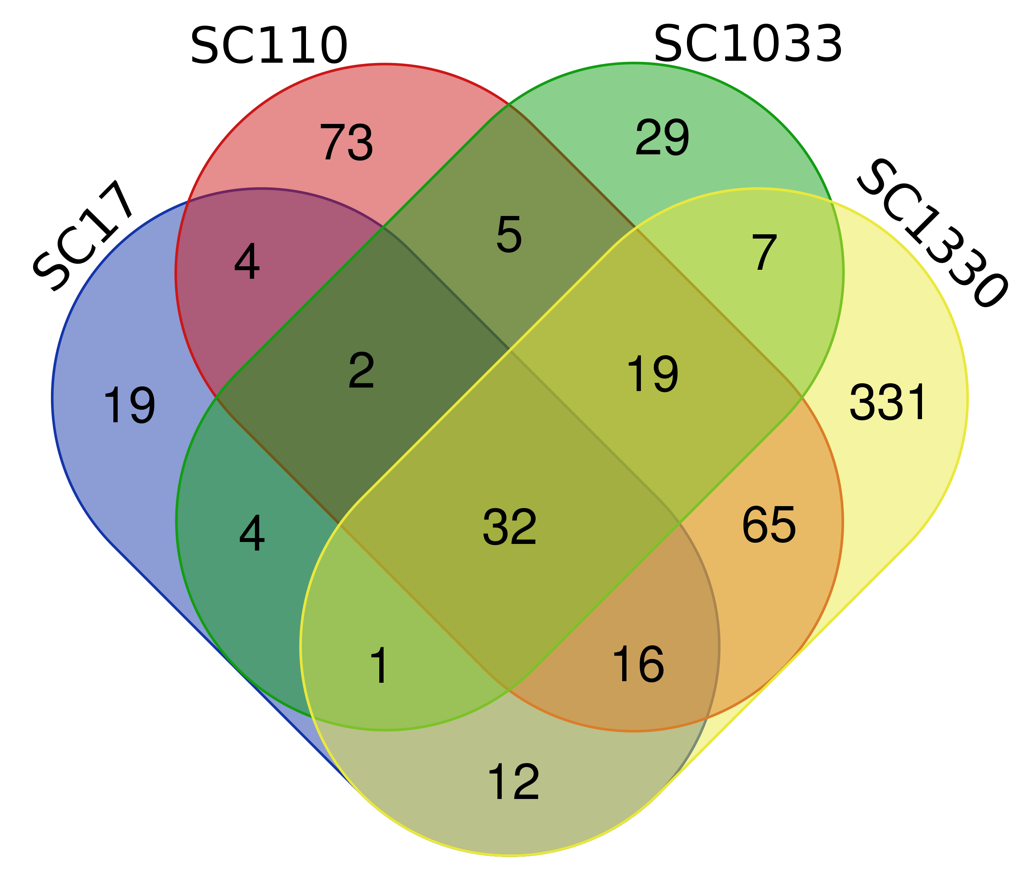


**Supplementary Figure 3.** Venn diagram of up-regulated genes expressed in the *C. sublineola* transcriptome in sorghum genotypes SC17, SC110, SC1033, and SC1330 across time points in inoculated versus mock-inoculated samples. Figure made with <https://bioinformatics.psb.ugent.be/webtools/Venn/>.


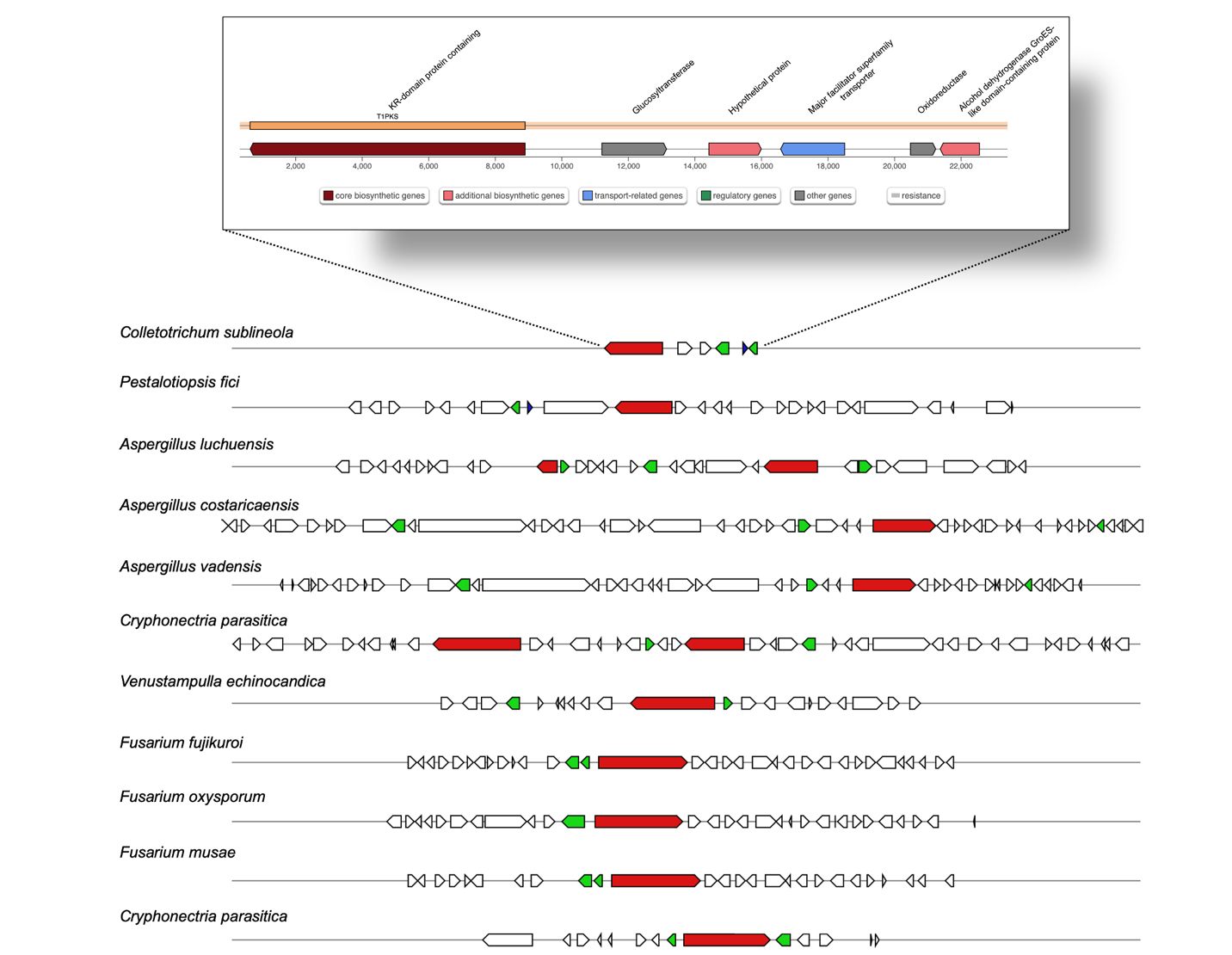


**Supplementary Figure 4.** The organization of the predicted biosynthetic gene cluster (BGC) in *C. sublineola* identified from the up-regulated gene *CSUB01_10385* encoding a KR-domain containing protein (red) in a cluster with other biosynthetic genes (green) and a gene encoding a transporter (blue). Fungal species containing similar BGCs are displayed. Figure created using antiSMASH (Blin et al., 2023).
